# Reduced GABAergic inhibition and impaired synapse elimination by neuroligin-2 deletion from Purkinje cells of the developing cerebellum

**DOI:** 10.3389/fncir.2025.1530141

**Published:** 2025-03-14

**Authors:** Esther Suk King Lai, Naofumi Uesaka, Taisuke Miyazaki, Kouichi Hashimoto, Masahiko Watanabe, Masanobu Kano

**Affiliations:** ^1^Department of Neurophysiology, Graduate School of Medicine, The University of Tokyo, Tokyo, Japan; ^2^Department of Cognitive Neurobiology, Graduate School of Medical and Dental Sciences, Institute of Science Tokyo, Tokyo, Japan; ^3^Department of Functioning and Disability, Faculty of Health Sciences, Hokkaido University, Sapporo, Japan; ^4^Department of Neurophysiology, Graduate School of Biomedical and Health Sciences, Hiroshima University, Hiroshima, Japan; ^5^Department of Anatomy, Hokkaido University Graduate School of Medicine, Sapporo, Japan; ^6^International Research Center for Neurointelligence (WPI-IRCN), The University of Tokyo, Tokyo, Japan; ^7^Advanced Comprehensive Research Organization (ACRO), Teikyo University, Tokyo, Japan

**Keywords:** synapse elimination, climbing fiber, Purkinje cell, cerebellum, neuroligin-2, Nlgn2_6_, inhibition, postnatal development

## Abstract

Functionally mature neural circuits are shaped during postnatal development by eliminating redundant synapses formed around birth. This process is known as synapse elimination and requires a proper balance of excitation and inhibition. Neuroligin-2 (NL2) is a postsynaptic cell adhesion molecule required for the formation, maintenance, and function of inhibitory synapses. However, how NL2 regulates synapse elimination during postnatal development is largely unknown. Here we report that the deletion of NL2 from Purkinje cells (PCs) in the cerebellum impairs the developmental elimination of redundant climbing fiber (CF) to PC synapses. In global NL2-knockout (KO) mice, GABAergic inhibition to PCs was attenuated and CF synapse elimination was impaired after postnatal day 10 (P10). These phenotypes were restored by the expression of NL2 into PCs of NL2-KO mice. Moreover, microRNA-mediated knockdown of NL2 specifically from PCs during development caused attenuated inhibition and impaired CF synapse elimination. In PCs innervated by “strong” and “weak” CFs, calcium transients elicited by “weak” CFs were enhanced in NL2-deficient PCs, suggesting that excess calcium signaling permits the survival of redundant “weak” CF synapses. We conclude that NL2 is crucial for maintaining inhibitory synaptic function and properly eliminating redundant CF synapses during postnatal development.

## Introduction

Neuroligins (NLs) are a family of synaptic cell adhesion molecules that localize at the postsynaptic membrane of excitatory and inhibitory synapses. By binding to presynaptic neurexins (NRXs), NLs form trans-synaptic complexes and mediate bi-directional signaling that organizes both presynaptic and postsynaptic protein compartments and regulates synapse formation, maturation, and function (Bottos et al., 2011; Bourne and Marchot, 2014; Lise and El-Husseini, 2006; Varoqueaux et al., 2006). Studies from mice lacking all NLs indicate a drastic change in the balance of glutamatergic and GABAergic/glycinergic transmission particularly in the respiratory brainstem regions (Varoqueaux et al., 2006). *In vivo* studies on mice with altered expression of NL isoforms further demonstrate that NLs do not simply regulate excitatory or inhibitory synapse formation but may act more finely to sway the ratio of excitation to inhibition. For instance, overexpression of NL1 in mice elevated the ratio of excitation to inhibition, altered the morphology of dendritic spines, impaired synaptic long-term potentiation, and caused learning deficits in the hippocampus (Dahlhaus et al., 2010). In contrast, transgenic mice with enhanced expression of NL2 exhibited altered synapse morphology, a shift in the synaptic balance toward inhibition, and behavioral abnormalities relevant to neurodevelopmental disorders (Hines et al., 2008). These studies suggest that the expression of distinct NLs at excitatory and inhibitory synapses helps maintain proper balance between excitation and inhibition (E/I balance) in neural circuits which may be essential for normal information processing in the brain. Given that the establishment of functional neural circuitry is dynamic and requires proper formation and refinement of synaptic connections, bi-directional signaling through NL-NRX complex should be crucial not only for synaptogenesis and the assembly of synaptic specializations but also for the maturation and maintenance of synapses. Thus, it is reasonable to hypothesize that changes in the expression of individual NLs and resultant alteration in the E/I balance may affect the refinement of neural circuits during early postnatal development.

Formation of functionally mature neural circuits is achieved by the elimination of redundant synaptic connections formed earlier during development and the strengthening of a subset of synapses that are thought to be necessary for brain function (Katz and Shatz, 1996; Lichtman and Colman, 2000; Nagappan-Chettiar et al., 2023; Purves and Lichtman, 1980). This process is termed synapse elimination and is widely observed in various regions of the developing nervous system. In the cerebellum, massive elimination occurs at climbing fiber (CF) to Purkinje cell (PC) synapses during postnatal development and this event is regarded as a representative model to study synapse elimination (Crepel, 1982; Kano and Watanabe, 2019; Kano et al., 2018; Lohof et al., 1996). In the cerebellum of neonatal rodents, each PC receives glutamatergic excitatory inputs from multiple (more than five) CFs, the terminal branches of axons of neurons in the contralateral inferior olive. The multiple CFs innervating each PC have similar synaptic strengths in neonatal mice. A single CF selectively becomes stronger relative to the other CFs during the first postnatal week, and only the strongest CF extends its synaptic territory along PC dendrites from around postnatal day 9 (P9) and thereafter. In parallel, surplus CF synapses on the PC soma are eliminated from around P7 to P17, and most PCs become innervated by single strong CFs on their proximal dendrites (mono-innervation). We reported previously that CF synapse elimination was impaired in mice with heterozygous genetic deletion of the GABA-synthesizing enzyme GAD67 (GAD67^+/GFP^ mice) (Nakayama et al., 2012). We found that GABAergic inhibition from basket cells to PCs was diminished at P10-P12 and CF synapse elimination was impaired after P10 (Nakayama et al., 2012). These results indicate that reduced GABAergic inhibition in PCs during the second postnatal week impairs CF synapse elimination. Since NL2 is crucial for the formation, maturation, and function of GABAergic inhibitory synapses (Hines et al., 2008; Varoqueaux et al., 2004), we assume that NL2 is indispensable for maintaining inhibitory synapses in PCs and is involved in CF synapse elimination. Zhang et al. (2015) demonstrated that conditional deletion of NL2 in PCs resulted in reduced inhibitory synaptic transmission from basket cells and stellate cells to PCs without attenuation of excitatory synaptic transmission (Zhang et al., 2015), indicating an elevated E/I balance in NL2-deficient PCs. However, CF synapse elimination was untested in the PC-specific conditional NL2-knockout (KO) mice (Zhang et al., 2015). In our preliminary study in olivocerebellar coculture preparations *in vitro*, we found that RNAi-mediated knockdown of NL2 in PCs increased the average number of EPSC steps by CF stimulation, suggesting that NL2 is involved in CF synapse elimination (Uesaka et al., 2012).

The present study aimed to elucidate how deletion of NL2 in mouse PCs *in vivo* alters the E/I balance of synaptic transmission in PCs and affects CF synapse elimination. The results to be presented indicate that NL2 positively regulates GABAergic inhibition, maintains the E/I balance, and contributes to CF synapse elimination after P10 in the developing cerebellum.

## Materials and methods

### Animals

NL2-KO mice were purchased from The Jackson Laboratory (Maine, United States). Both wild-type and homozygote NL2-KO littermates of both sexes from the interbreeding of heterozygote pairs were used. In experiments with *in utero* electroporation, C57BL/6 mice were used (SLC, Japan). All experiments were conducted following the guidelines for the care and use of laboratory animals of the University of Tokyo, Hokkaido University, and the Japan Neuroscience Society.

### *In utero* electroporation

Pregnant C57BL/6 mice at embryonic day (E) 11.5 or E12.5 (SLC, Japan) were deeply anesthetized via intraperitoneal injection (50 μg/g) of sodium pentobarbital (Somnopentyl, Kyoritsu Seiyaku Co., Tokyo, Japan). One microliter of a plasmid DNA (1.5 μg/μL) in phosphate-buffered saline containing fast green was injected into the fourth ventricle of embryonic brains. When we transfected two or three different plasmid DNA constructs into PCs, we first prepared individual constructs in phophosphate-buffered saline containing fast green at the concentration of 1.5 μg/μL. We took 1 μL of the individual DNA-containing solutions and mixed them. Then we injected 1 μL of the mixed solution into the fourth ventricle of embryonic brains. The embryo was held in uterus with a forceps-type electrode, and electrical pulses (40 V, with a duration of 50 ms, at intervals of 950 ms per cycle) were delivered five times with an electroporator (Nepa Gene, Japan). After electroporation, the uterus was repositioned in the abdominal cavity, the abdominal wall and skin were closed, and the embryos were allowed to continue developing normally.

### Vector construction

Plasmid vectors were designed for PC-specific expressions under the control of a truncated L7 promoter (pCL20c-L7). These vectors were constructed to express GFP and/or microRNA (miRNA) directed against NL2 or P/Q-type voltage-gated calcium channels (P/Q-VDCC) under the control of a truncated L7 promoter. The plasmid with mouse NL2 construct was obtained from Addgene (plasmid 15246) (Chih et al., 2006). The NL2 sequence was cloned into the pCAGGS vector. Engineered microRNAs targeting NL2 were designed according to the BLOCK-iT Pol II miR RNAi Expression Vector Kit guidelines (Invitrogen), using the following sequences: 5’-TGCTGTGTACATCCTGGTCCACTAGCGTTTTGGCCACTGACTGACGCTAGTGGCAGGATGTACA-3′ and 5’-CCTGTGTACATCCTGCCACTAGCGTCAGTCAGTGGCCAAAACGCTAGTGGACCAGGATGTACAC-3′. For P/Q-VDCC, we used the same microRNA sequence as reported by Mikuni et al. (2013).

### Electrophysiology

Mice at P4 to P35 were decapitated following CO_2_ anesthesia, and brains were rapidly removed and placed in chilled normal ACSF (0–4°C) containing (in mM) 125 NaCl, 2.5 KCl, 2CaCl_2_, 1MgSO_4_, 1.25 NaH_2_PO_4_, 26 NaHCO_3_, and 20 glucose, bubbled with 95% O_2_ and 5% CO_2_ (pH 7.4). Parasagittal cerebellar slices (250 μm) were prepared by using a vibratome slicer (VT-1200S, Leica, Germany). Whole-cell patch-clamp recordings were made from visually identified PCs by using an upright microscope (BX50W1, Olympus, Japan). Internal solution composed of (in mM): 60 CsCl, 10 D-gluconate, 20 TEA-Cl, 20 BAPTA, 4 MgCl_2_, 4 ATP, 0.4 GTP, and 30 HEPES (pH 7.3, adjusted with CsOH) for recording excitatory postsynaptic currents (EPSCs) and 124 CsCl, 10 HEPES, 10 BAPTA, 1 CaCl2, 4.6 MgCl_2_, 4 ATP, 0.4 GTP (pH 7.3, adjusted with CsOH) for recording inhibitory postsynaptic currents (IPSCs). Picotoxin (100 μM) was added to block inhibitory synaptic transmission for recording stimulation-evoked EPSCs. Picotoxin (100 μM) and tetrodotoxin (0.5 μM) were added for recording miniature EPSCs (mEPSCs). NBQX (10 μM), _R_-CPP (5 μM) and tetrodotoxin (0.5 μM) were added for recording miniature IPSCs (mIPSCs). Stimulation pipettes (5–10 μm tip diameter) were filled with the normal ACSF and used to apply square pulses for focal stimulation (duration of 100 ms, amplitude of 0 V to 100 V). CFs were stimulated in the granular layer around the PC soma under investigation. All electrophysiological recordings were performed at 32°C. Ionic currents were recorded with an EPC10 patch clamp amplifier (HEKA). Online data acquisition and offline data analysis were performed using PULSE and PULSE FIT software (HEKA) or Mini analysis Program (ver. 4.0.1, Synaptosoft Inc.). Liquid junction potential was corrected. All drugs were obtained from Tocris.

To quantitatively evaluate the disparity among the amplitudes of multiple CF-mediated EPSCs (CF-EPSCs) in individual PCs, we calculated the disparity ratio and the disparity index as shown previously (Hashimoto and Kano, 2003).

Disparity ratio= $\frac{\left(\frac{A_{1}}{A_{N}}+\frac{A_{2}}{A_{N}}+\cdots +\frac{A_{N-1}}{A_{N}}\right)}{\left(N-1\right)\text{.}}$

Disparity index = $\frac{S.D.}{M}$


$$M=\sum \frac{\mathrm{Ai}}{N}\left(i=1,2,3,\ldots N;N>2\right)$$

S.D. = $\sqrt{\sum \frac{{\left(A_{i}-M\right)}^{2}}{N-1}}$

To calculate the disparity ratio and disparity index, the amplitudes of individual CF-EPSCs in a given PC with multiple CF innervation were measured at the same holding potential and numbered in the order of their amplitudes (A1, A2, …, AN, N ≥ 2; N is the number of CFs innervating a given PC. AN represents the largest CF-EPSC) (Hashimoto and Kano, 2003). The smaller the difference in the amplitude between AN and other weaker CF-EPSCs, the larger the value of disparity ratio. If all CFs innervating a PC exhibit similar amplitudes of CF-EPSCs, the disparity ratio approaches 1. The disparity index is the coefficient of variation for all CF-EPSC amplitudes recorded in a PC (Hashimoto and Kano, 2003). The larger the difference in the amplitude of CF-EPSCs, the greater the value of the disparity index.

### Calcium imaging

Vermal cerebellar slices (250 μm thickness) were prepared from mice at P10 to P13. Recording pipettes were filled with internal solutions containing (in mM): 135 K-gluconate, 10 Na-gluconate, 5 KCl, 0.5 EGTA, 10 HEPES, 4 Mg-ATP, 0.4 Na_3_-GTP (pH 7.3, adjusted with NaOH) and 0.1 OGB-1, which was used for imaging CF-induced calcium signals. After establishing whole-cell recording configurations, PCs were loaded for at least 20 min with the calcium indicator OGB-1. In the normal ACSF without blockers of excitatory or inhibitory synaptic transmission, fluorescence images were acquired at 30 Hz using a high-speed confocal laser scanning microscope (CSU22, Yokogawa). The calcium signals were obtained from the soma or proximal dendritic regions of PCs. The calcium-dependent fluorescence signals from selected regions of interest (ROI) were background subtracted and expressed as increases in fluorescence divided by the prestimulus fluorescence values (ΔF/F_0_) using ImageJ.^1^ The calcium imaging was performed at 32°C.

### Morphological analysis

For immunofluorescence, mice were deeply anesthetized with pentobarbital (100 mg/g of body weight, i.p.), perfused with 4% paraformaldehyde in 0.1 M sodium phosphate buffer, pH 7.2, and processed for parasagittal microslicer sections (50 μm in thickness; VT1000S; Lecia, Nussloch, Germany). After permeabilization and blocking for nonspecific binding of antibodies, the sections were incubated overnight with affinity-purified primary antibodies with 2% normal donkey serum and 0.3% Triton X-100 in phosphate-buffered saline (PBS) for 48 h at 4°C. Subsequently, the sections were rinsed in PBS and then incubated for 2 h at room temperature with a mixture of fluorochrome-conjugated secondary antibodies (species-specific indocarbocyanine (Cy3)- or indodicarbocyanine (Cy5)-conjugated secondary IgG; 1:200, Jackson ImmunoResearch, West Grove, PA) in the dark. Incubation was finally stopped by washing three times with PBS. After washing, the sections were mounted in the mounting medium (DAKO, Carpinteria, CA, United States) and then examined under a laser scanning confocal microscope (FV1000, Olympus).

For the morphological analysis of CF innervation, mice were anesthetized with chloral hydrate (350 mg/kg of body weight, i.p.). A glass pipette filled with 2–3 μL of 10% solution of dextran Alexa Fluor-594 (DA-594, Invitrogen) in PBS was inserted stereotaxically into the inferior olive by the dorsal approach, as described previously (Miyazaki et al., 2004; Miyazaki and Watanabe, 2011). Tracers were injected by air pressure (Pneumatic Picopump; World Precision Instruments). After 4 days of survival, mice were fixed by transcardial perfusion. For combined labeling by tracer and immunofluorescence, DA-594-labeled microslicer sections were incubated with a mixture of calbindin and VGluT2 antibodies followed by 2 h incubation with a mixture of Alexa 488- (Invitrogen) and Cy5-labeled species-specific secondary antibodies. Images of triple labeling were taken with a confocal laser-scanning microscope (FV1000, Olympus).

### Western blot

Lysates of cerebella from 2-month-old NL2-KO and wild-type mice were prepared using standard protocols. Briefly, cerebella were lysed with RIPA buffer (125 mM Tris–HCl, pH 6.8; 10% Mercaptoethanol, 0.004% Bromophenol Blue, 10% Sucrose, and 4% SDS; Nacalai tesque, Kyoto, Japan), and then the lysates were left on ice for 30–60 min and centrifuged at 10,000 g at 4°C for 10 min. Equal amounts of lysates were resolved on 10% polyacrylamide-SDS gel by SDS-PAGE (at 196 V and 40 mA for 90 min) and transferred to polyvinylidene difluoride membranes. The blot was performed at 25 V and 100 mA for 2 h. Subsequently, these membranes were probed with the indicated primary antibodies overnight at 4°C, followed by incubation with HRP-conjugated secondary antibodies for 1 h at room temperature. Proteins were visualized by HRP reaction using ECL SuperSignal West Pico kit (Thermo Scientific, Waltham, MA, USA). Data acquisition and analysis were performed using an ImageQuant LAS 4000 image analyzer (GE Healthcare, Chicago, IL, United States).

### Antibodies

Affinity-purified primary antibodies raised against the following molecules (host species, final concentration) were used: anti-NL2 (rabbit, 1 μg/mL; Synaptic Systems, Germany), anti-NL3 (rabbit, 1 μg/mL; Synaptic Systems, Germany), anti-calbindin (goat, 1 mg/mL) (Miura et al., 2006), anti-vesicular glutamate transporter 1 (VGluT1) (1 mg/mL, Frontier Institute), anti-vesicular glutamate transporter 2 (VGluT2) (1 mg/mL, Frontier Institute), anti-VGAT (rabbit, 1 mg/mL) (Miura et al., 2006), anti-GABA_A_ receptor α1 (guinea pig, 1:1000; Synaptic Systems, Germany), anti-GABA_A_ receptor α2 (guinea pig, 1:1000; Synaptic Systems, Germany), anti-PSD 95 (rabbit, 1:250; Invitrogen), and anti-Gephyrin (mouse, 1:1000, Synaptic Systems, Germany).

### Statistics

All statistical values are presented as mean ± SD unless indicated otherwise. First, normality was checked for individual datasets by using the Shapiro–Wilk test. Since normality was often not assured, we employed nonparametric statistical tests in the present study. We used the Mann–Whitney U test to compare two independent datasets, and the Kruskal-Wallis test followed by Dunn’s test for multiple comparisons or the Mann–Whitney test with False Discovery Rate (FDR) correction using the Benjamini-Hochberg procedure for multiple comparisons. We used the Kolmogorov–Smirnov test for datasets with cumulative distributions. Differences were considered statistically significant if the *p*-value was smaller than 0.05. All statistical analyses were conducted with GraphPad Prism 10 Software (GraphPad Software, La Solla, CA, USA).

## Results

### Reduced inhibitory synaptic transmission to PCs in NL2-KO mice

**Figure 1:**
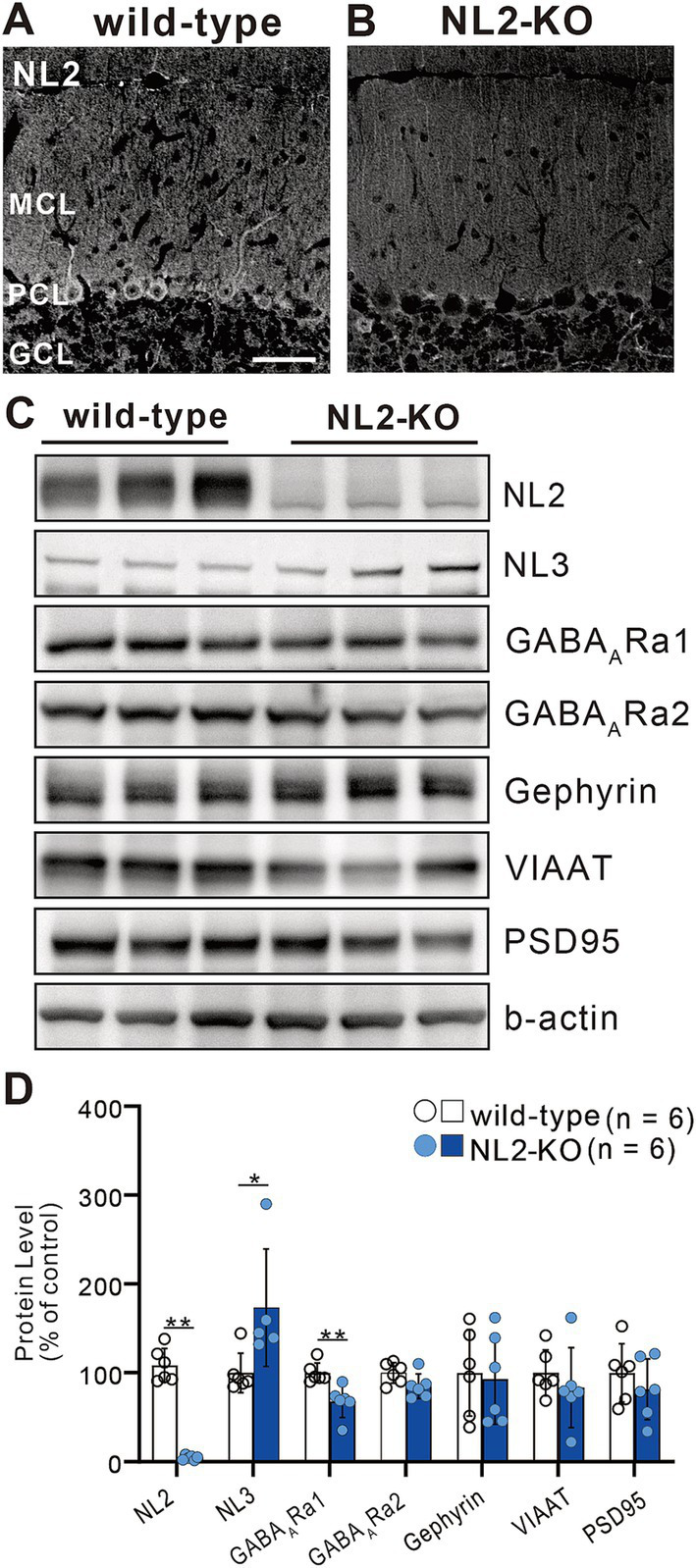
Elevated expression of NL3 in the developing cerebellum of NL2-KO mice. **(A,B)** Immunohistochemistry for NL2 in the cerebellar cortex of a wild-type **(A)** and an NL2-KO **(B)** mouse at adult age. Note that NL2 immunoreactivity is highly localized in PCs and interneurons (arrowheads) in wild-type mice, but almost absent in NL2-KO mice. GCL, granule cell layer; MCL, molecular layer; PCL, Purkinje cell layer. Scale bar, 50 μm. **(C,D)** Representative immunoblots **(C)** and quantification **(D)** of total protein levels in cerebellar homogenates from wild-type and NL2-KO mice (*n* = 6/group). Protein expression of NL3 is significantly increased while that of GABA_A_ receptor α1 subunit is significantly decreased in the cerebella of NL2-KO mice compared to those of wild-type mice. All data are shown as means ± SD. *p < 0.05 and **p < 0.01 by Mann–Whitney U test.

We first checked whether global knockout (KO) of NL2 affected the expression of synaptic proteins in the cerebellum by quantitative western blot analysis of cerebellar lysates (Figure 1). We found that the expression of NL2 was almost undetectable (Figures 1A,B) (> 90% decrease, *p* = 0.002, Mann–Whitney U test) and that the expression of GABA_A_ receptor α1 was moderately decreased to approximately 33% (*p* = 0.002, Mann–Whitney U test) in NL2-KO mice compared to those in wild-type mice (Figures 1C,D). In contrast, we observed that the expression of NL3 was significantly increased in NL2-KO mice compared to that in wild-type mice (73% increase, *p* = 0.017, Mann–Whitney U test), presumably because of compensation for the lack of NL2 (Figures 1C,D). We found no significant difference in the expression of GABA_A_ receptor α2 (*p* = 0.093, Mann–Whitney U test), gephyrin (*p* = 0.937, Mann–Whitney U test), vesicular inhibitory amino acid transporter (VIAAT; *p* = 0.24, Mann–Whitney U test), or PSD95 (*p* = 0.589, Mann–Whitney U test) (Figures 1C,D). These results suggest that GABA_A_ receptor-mediated inhibitory synaptic transmission may be attenuated in NL2-KO mice.

**Figure 2:**
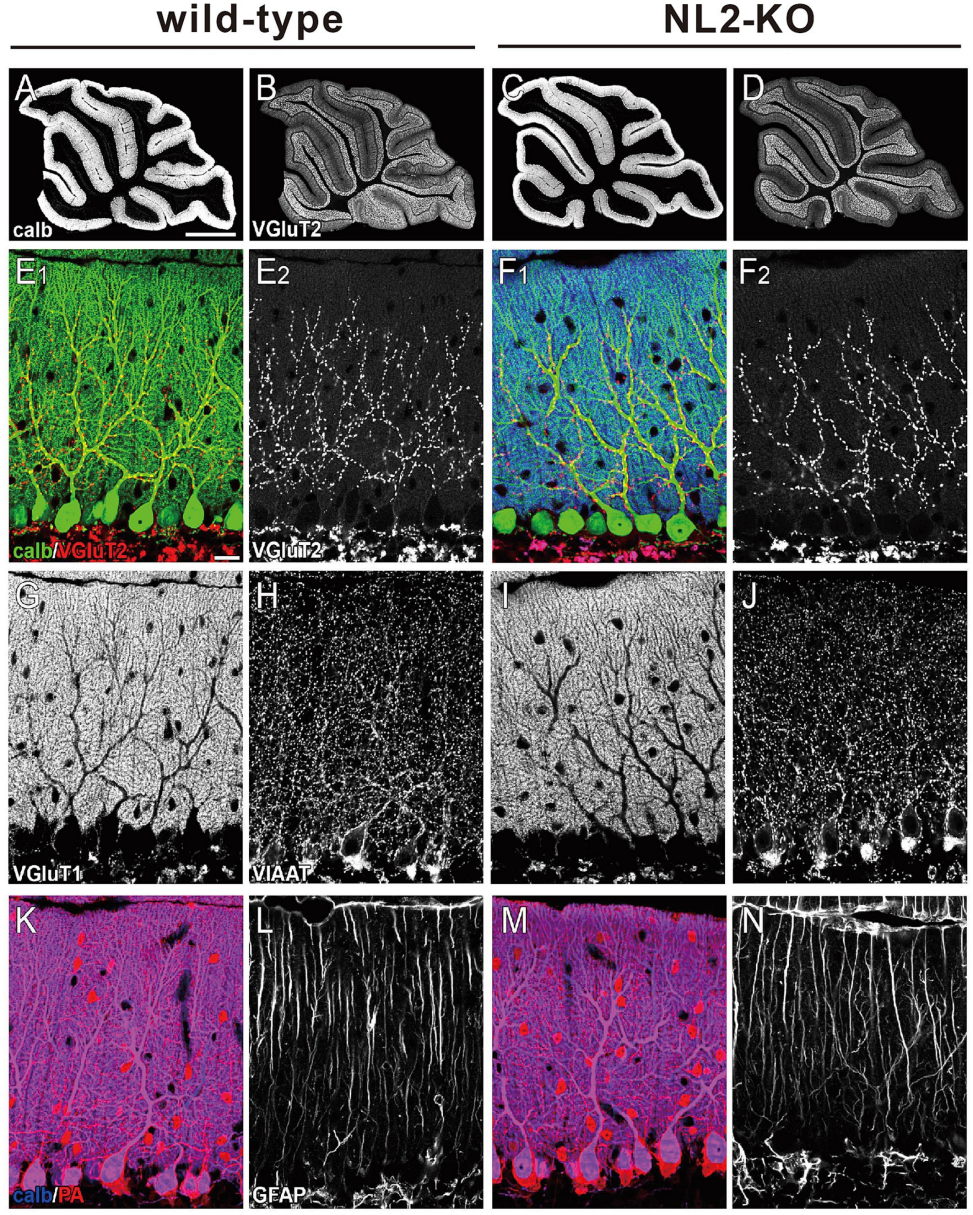
Gross anatomy of the cerebellum of wild-type and NL2-KO mice. **(A-D)** Immunohistochemistry for calbindin **(A,C)** and VGluT2 **(B,D)** in the cerebellar vermis of a wild-type and an NL2-KO mouse at an adult age. Scale bar in **A**, 1 mm. **(E–N)** Immunohistochemistry for a PC marker, calbindin (calb, **E,F,K,M**), a CF presynaptic terminal marker, vesicular glutamate transporter 2 (VGluT2, **E,F**), a PF presynaptic terminal marker, vesicular glutamate transporter 1 (VGluT1, **G,I**), molecular layer inhibitory interneuron markers, vesicular inhibitory amino acid transporter (VIAAT, **H,J**) and parvalbumin (PA, **K,M**), and a Bergmann astrocyte marker, glial fibrillary acid protein (GFAP, **L,N**) in adult wild-type and NL2-KO mice. Scale bar in **E**_**1**_, 20 μm.

We then checked the gross anatomy of the cerebellum and the morphology of its major cellular and synaptic components by immunostaining for their markers. We found that the foliation and layer structure of the cerebellum and the morphology and density of Purkinje cell (PC) immunostained for its marker calbindin were normal in NL2-KO mice (Figures 2A–D). We found no significant differences between the genotypes in the pattern and density of immunofluorescence for a parallel fiber (PF) terminal marker, type 1 vesicular glutamate transporter (VGluT1), and that for a climbing fiber (CF) terminal marker, type 2 vesicular glutamate transporter (VGluT2) (Figures 2E–G,I). Immunofluorescence for an inhibitory terminal marker, VIAAT, and that for an inhibitory interneuron marker, parvalbumin (PA), were similar between wild-type and NL2-KO mice (Figures 2H,J–K,M). Furthermore, no appreciable difference was noted between the genotypes in the morphology of Bergmann glia immunostained for its marker glial fibrillary acidic protein (GFAP) (Figures 2L,N).

**Figure 3:**
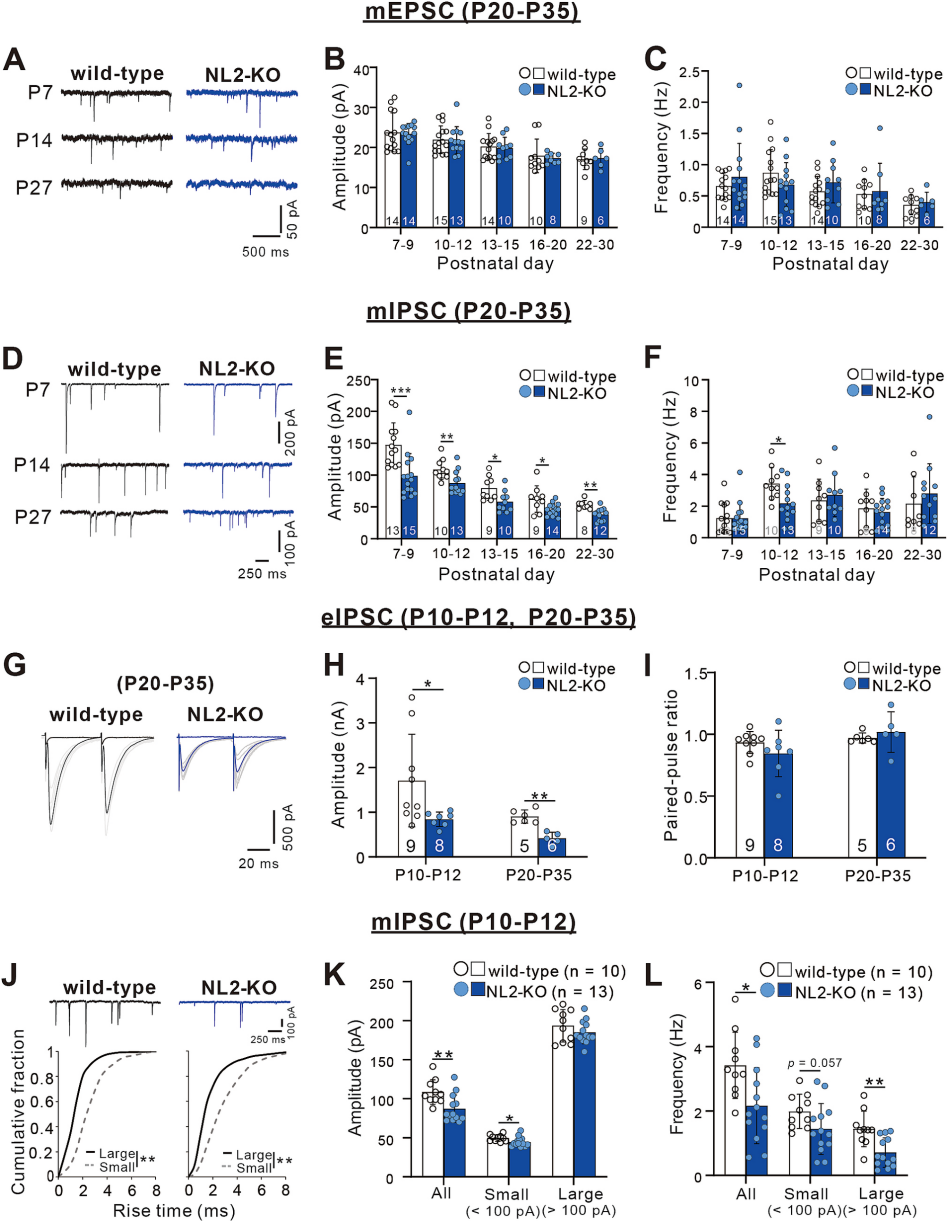
Attenuated inhibitory synaptic transmission in PCs of NL2-KO mice. **(A)** Representative traces of mEPSC recorded in PCs of wild-type (left) and NL2-KO (right) mice at P7, P14, and P27 with the holding potential (Vh) of −70 mV in the presence of 1 μM TTX, 0.1 mM Picotoxin. Scale bars, 500 ms and 50 pA. **(B,C)** Summary bar graphs and individual data distributions for the mEPSC amplitude **(B)** and frequency **(C)** during postnatal development. **(D)** Representative traces of mIPSC recorded in PCs of wild-type (left) and NL2-KO (right) mice at P7, P14, and P27 in the presence of 1 μM TTX, 10 μM NBQX and 5 μM _R_-CPP. Vh = -70 mV. Scale bars, 250 ms and 100 pA for P7 and 50 pA for P14 and P27. **(E,F)** Summary bar graphs and individual data distributions for the mIPSC amplitudes **(E)** and frequency **(F)** during postnatal development. **(G)** Representative traces of eIPSC recorded in a wild-type (left) and an NL2-KO (right) mouse during P20-P35 in the presence of 10 μM NBQX and 5 μM _R_-CPP. Vh = -70 mV. Scale bars, 20 ms and 500 pA. **(H,I)** Summary bar graphs and individual data distributions for the eIPSC amplitudes **(H)** and the paired-pulse ratio at 50 ms intervals **(I)** recorded during P10-P12 and P20-P35. **(J)** (upper panel) Representative traces of mIPSC recorded in a wild-type (left) and an NL2-KO (right) mouse during P10-P12 in the presence of 1 μM TTX, 10 μM NBQX and 5 μM _R_-CPP. (Lower panel) Cumulative fractions of the rise time of small (< 100 pA; grey dotted line) and large (> 100 pA; black line) mIPSCs in wild-type and NL2-KO mice. Data were obtained from P10–P12 mice. **p < 0.001 by Kolmogorov–Smirnov test for both genotypes. Vh = −70 mV. Scale bars, 250 ms and 100 pA. **(K,L)** Summary bar graphs and individual data distributions showing the mIPSC amplitude **(H)** and frequency **(I)** for all, small and large events in wild-type and NL2-KO mice. Data in **B,C,E,F,H,I,K,L** are shown as means ± SD. *p < 0.05; **p < 0.01; ***p < 0.001 by the Mann–Whitney test with FDR correction using the Benjamini-Hochberg procedure. The number of recorded cells is indicated within each column.

Zhang et al. (2015) reported previously that PC-specific double NL2- and NL3-KO and single NL2-KO severely impaired inhibitory synaptic transmission in PCs at P21-P25 (Zhang et al., 2015). We, therefore, examined whether synaptic transmission is altered in the PCs of our global NL2-KO mice. We performed whole-cell recordings from PCs in acute cerebellar slices and measured miniature excitatory postsynaptic currents (mEPSCs) and miniature inhibitory postsynaptic currents (mIPSCs) (Yamasaki et al., 2006). We found no significant difference in the amplitude (P7-9: *p* = 0.563; P10-12: *p* = 0.928; P13-15: *p* = 0.796; P16-20: *p* = 0.315; P22-30: *p* = 0.776, Mann–Whitney test with FDR correction using the Benjamini-Hochberg procedure) and the frequency (P7-9: *p* = 0.743; P10-12: *p* = 0.254; P13-15: *p* = 0.312; P16-20: *p* = 0.848; P22-30: *p* = 0.864, Mann–Whitney test with FDR correction using the Benjamini-Hochberg procedure) of mEPSCs between wild-type and NL2-KO PCs throughout the age ranges during postnatal development (Figures 3A–C). In contrast, the amplitude of mIPSCs was consistently smaller in NL2-KO mice than in wild-type mice throughout postnatal development from P7-P9 to P22-P30 (P7-9: *p* < 0.001; P10-12: *p* = 0.008; P13-15: *p* = 0.017; P16-20: *p* = 0.028; P22-30: *p* = 0.001, Mann–Whitney test with FDR correction using the Benjamini-Hochberg procedure; Figures 3D,E). The frequency of mIPSCs was lower at P10-P12 (wild-type: 3.42 ± 1.029 Hz; NL2-KO: 2.17 ± 1.18 Hz, *p* = 0.015) but showed no difference in the other age ranges between wild-type and NL2-KO PCs (P7-9: *p* = 0.683; P13-15: *p* = 0.661; P16-20: *p* = 0.688; P22-30: *p* = 0.427, Mann–Whitney test with FDR correction using the Benjamini-Hochberg procedure; Figure 3F). These results indicate that GABAergic inhibition to PCs is attenuated in NL2-KO mice particularly from P10 to P12 when massive formation of inhibitory synapses occurs from molecular layer interneurons to PCs (Yamanaka et al., 2004). We also recorded IPSCs from PCs elicited by extracellular stimulation (eIPSCs) at both P10-P12 and P20-P35 (Figures 3G–I). We observed a significant decrease in the eIPSC amplitude (~50%, Figure 3H) in NL2-KO PCs (P10-P12: *p* = 0.018, and P20-P35: *p* = 0.004, Mann–Whitney test with FDR correction using the Benjamini-Hochberg procedure), with no alteration in the paired-pulse ratio (P10-P12: *p* = 0.21, and P20-P35: *p* = 0.247, Mann–Whitney test with FDR correction using the Benjamini-Hochberg procedure; Figure 3I); suggesting that the reduction of the IPSC amplitude is caused by postsynaptic changes.

We further classified mIPSCs into two categories (small and large) at the amplitude of 100 pA and scrutinized their kinetics at P10 to P12 (Nakayama et al., 2012). The rise time of large mIPSCs was significantly faster than that of small mIPSCs (Figure 3J), supporting that large mIPSCs arise mainly from synapses on the soma (presumably from basket cells) and small mIPSCs arise mainly from synapses on dendrites (presumably from stellate cells) to PCs (Nakayama et al., 2012). We found that the amplitude of small mIPSCs was slightly smaller (wild-type: 49.72 ± 4.124 pA, *n* = 10; NL2-KO: 44.7 ± 6.195 pA, *n* = 13, *p* = 0.018, Mann–Whitney test with FDR correction using the Benjamini-Hochberg procedure), whereas the frequency of large mIPSCs was significantly reduced (wild-type: 1.44 ± 0.549 Hz, *n* = 10; NL2-KO: 0.71 ± 0.457 Hz, *n* = 13, *p* = 0.002, Mann–Whitney test with FDR correction using the Benjamini-Hochberg procedure) in NL2-KO mice than in wild-type mice (Figures 3K,L). These results suggest that inhibitory synaptic inputs from basket cells were reduced particularly at P10-P12 in PCs of NL2-KO mice. Taken together, the E/I balance of PCs is considered to be shifted to enhanced excitation in NL2-KO mice compared with wild-type mice.

### Persistent multiple CF innervation in PCs of mature NL2-KO mice

**Figure 4:**
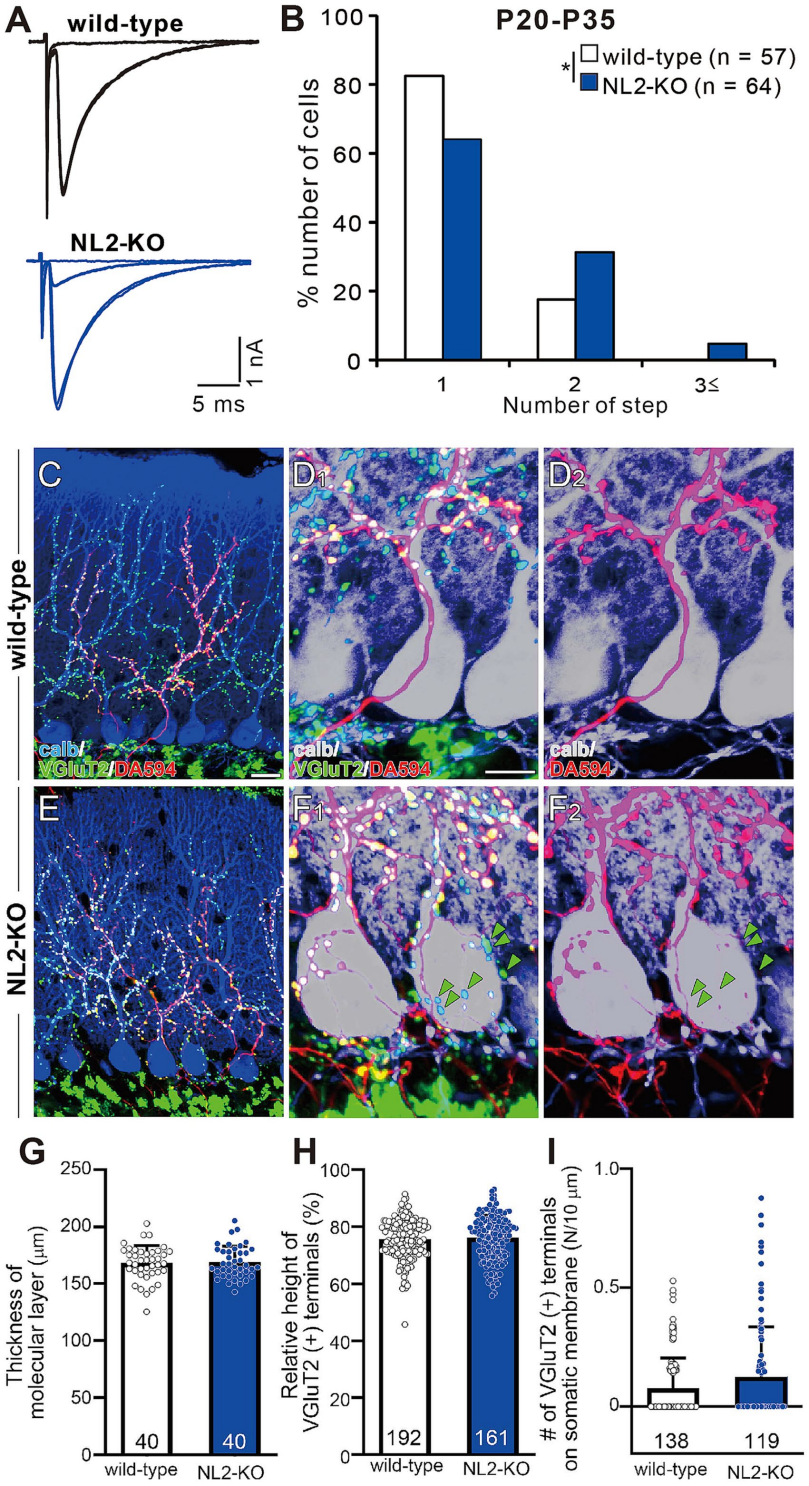
Persistent multiple CF innervation in PCs of mature NL2-KO mice. **(A)** Representative traces of CF-EPSC in a wild-type and an NL2-KO PC during P21 to P35. Vh = −10 mV. Scale bars, 5 ms and 1 nA. **(B)** Frequency distribution for the number of CF-EPSC steps in each PC for wild-type (open columns, *n* = 57) and NL2-KO (blue columns, *n* = 64) mice during P21-P35. *p < 0.05; Mann–Whitney *U* test. **(C–F)** Immunostaining for calbindin (blue), anterogradely labeled CFs (DA594, red), and VGluT2 (green) in a wild-type **(C,D1)** and an NL2-KO **(E,F1)** mouse at P28. **(D1,F1)** are the magnified views of **(C,E)**, respectively. **(D2,F2)** are the double immunostaining images for calbindin (blue) and DA594 (red) in a wild-type **(D2)** and an NL2-KO **(F2)** mouse. Note that VGluT2 puncta associated with DA594and those without DA594 (green arrowheads) were frequently found on NL2-KO PC somata. Scale bars, 20 μm for **C,E**, 10 μm for **D,F**. **(G)** Summary bar graph and individual data distributions showing the molecular layer thickness for wild-type (open column, *n* = 40 points from 3 mice) and NL2-KO (blue column, *n* = 40 points from 3 mice) cerebella. The vertical length from the basal tip of PC dendrite to the pial surface was measured at randomly selected 40 points in cerebellar sections from 3 mice of each genotype. **(H)** Relative height of VGluT2-labeled CF terminals in the molecular layer for wild-type (open column, *n* = 192) and NL2-KO (blue column, *n* = 161) cerebella. The distance between the highest VGluT2 signal on the PC’s dendritic arbor and the top of the PC soma was measured in each PC and the value was divided by the thickness of the molecular layer. **(I)** Number of VGluT2-labeled CF terminals on the somatic membrane for wild-type (open column, *n* = 138) and NL2-KO (blue column, *n* = 119) PCs. Data in **G–I** are shown as means ± SD. Mann–Whitney U test. The total number of cells is indicated within each column.

We examined whether the postnatal development of CF to PC synapses was affected by the loss of NL2. We recorded whole-cell membrane currents from PCs in acute cerebellar slices and stimulated CFs in the granule cell layer to induce CF-mediated excitatory postsynaptic currents (CF-EPSCs). We systematically moved the location of the CF stimulation pipette and gradually increased the stimulus intensity at each position. We estimated the number of CFs innervating the recorded PC from discrete CF-EPSC steps elicited in that PC (Hashimoto et al., 2009a; Hashimoto and Kano, 2003; Hashimoto et al., 2011). In the majority of wild-type PCs (Figure 4A), a clearly discernible CF-EPSC was elicited in an all-or-none fashion, indicating that such PCs were innervated by single CFs. By contrast, in NL2-KO mice aged from P20 to P35, only 60% of PCs were innervated by single CFs. The frequency distribution of PCs against the number of CF-EPSC steps demonstrates that a significantly higher proportion of PCs were innervated by multiple CFs in NL2-KO mice than in wild-type mice (Figure 4B, *p* = 0.029, Mann–Whitney U test). In both genotypes, each PC was either mono-innervated by a strong CF (termed “CF-mono”) or multiply innervated by a strong CF plus one or two weaker CFs (termed “CF-multi-S” and “CF-multi-W,” respectively) (Hashimoto et al., 2009a; Hashimoto and Kano, 2003). We compared the kinetics of these three types of CF inputs between wild-type and NL2-KO mice. We found no significant difference in the amplitude, the 10-90% rise time, and the paired-pulse ratio of CF-EPSCs (Supplementary Table S1), although the decay time constant for CF- mono and that for CF-multi-S were slightly shorter in NL2-KO PCs. These results suggest that the deletion of NL2 impaired CF synapse elimination with little effect on the basic properties of CF-EPSCs. Since proper PF synaptogenesis and normal PF to PC synaptic transmission are prerequisites for developmental CF synapse elimination (Hashimoto et al., 2001; Hashimoto et al., 2009b; Hirai et al., 2005; Ichikawa et al., 2002), we examined the nature of PF-mediated EPSCs in PCs of NL2-KO and wild-type mice during P22–P30. No difference was noted between the two mouse strains in the input/output relations and the extent of paired-pulse facilitation (Supplementary Figure S1). These results indicate that PF to PC synapses function normally in NL2-KO mice and suggest that the persistent multiple CF innervation of NL2-KO PCs does not result from impaired PF to PC synaptic function.

To examine CF innervation of PCs morphologically, we stained a subset of CFs by injecting a small amount of an anterograde tracer, Dextran Alexa Fluor 594 (DA594), into the inferior olive and then performed triple fluorescent labeling for calbindin (a PC marker), VGluT2 (a CF terminal marker), and DA594 (Miyazaki et al., 2004; Miyazaki and Watanabe, 2011). In both wild-type and NL2-KO mice, DA594-positive CFs followed the proximal dendrites of PCs and climbed up to around 80% of the molecular layer (Figures 4C,D2,E,F2). Presynaptic terminals of these DA594-labelled CFs along PC dendritic trees mostly overlapped with VGluT2 immunoreactivity (Figures 4C,D1,E,F1). We also confirmed that the molecular layer thickness (Figure 4G; wild-type: 168.2 ± 2.4 μm; NL2-KO: 168.9 ± 2.2 μm; *p* = 0.8277, Mann–Whitney U test) and the relative height of VGluT2-positive CFs to the molecular layer thickness (Figure 4H; wild-type: 75.5% ± 0.5%; NL2-KO: 76.1% ± 0.6%; *p* = 0.4279, Mann–Whitney U test) were normal in NL2-KO mice. These results show that dendritic translocation of single strong CF is normal in NL2-KO mice. Although no significant difference was found in the density of vGluT2-positive terminals on the soma between wild-type and NL2-KO mice (Figure 4I, wild-type: 0.077 ± 0.011; NL2-KO: 0.125 ± 0.02, *p* = 0.2543, Mann–Whitney U test), DA594-labeled/VGluT2-positive terminals and DA594-unlabeled/VGluT2-positive terminals were frequently colocalized on the PC soma of NL2-KO mice (Figure 4F1). These results demonstrate that multiple CF innervation in NL2-KO PCs reflects the presence of redundant synapses on the soma arising from CFs distinct from the main CF innervating the dendrites of the same PC.

### CF synapse elimination is impaired after P10 in NL2-KO mice

**Figure 5:**
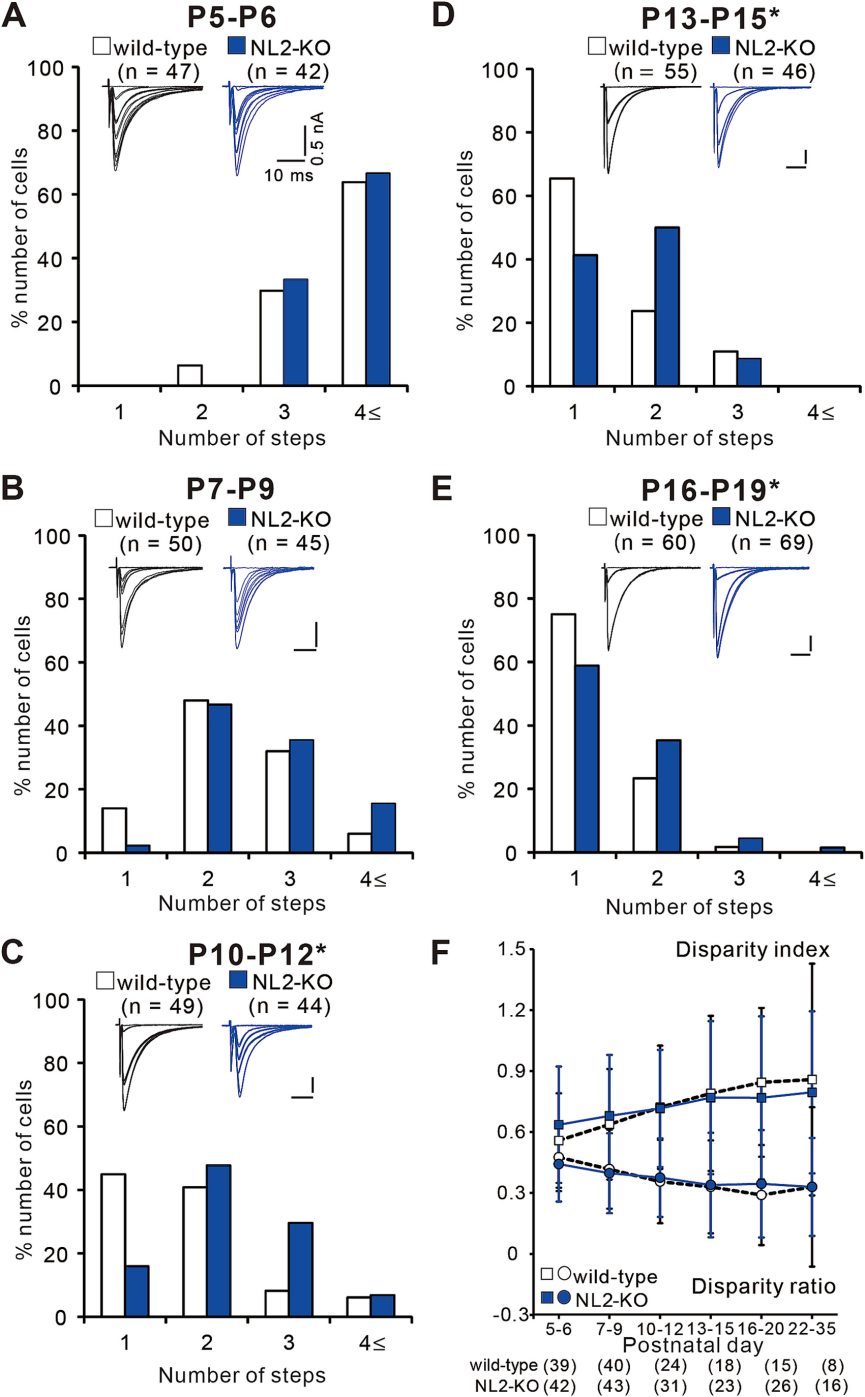
Impaired CF synapse elimination after P10 in NL2-KO mice. **(A–E)** Sample CF-EPSCs and frequency distribution histograms showing the number of CFs innervating each PC for wild-type (open columns) and NL2-KO (blue columns) mice at P5-P6 (**A**, *n* = 47 for wild-type and *n* = 42 for NL2-KO), P7-P9 (**B**, *n* = 55 for wild-type and *n* = 46 for NL2-KO), P10-P12 (**C**, *n* = 49 for wild-type and *n* = 44 for NL2-KO), P13-P15 (**D**, *n* = 55 for wild-type and *n* = 46 for NL2-KO), and P16-P19 (**E**, *n* = 60 for wild-type and *n* = 69 for NL2-KO). Vh = −70 mV **(A)** and − 10 mV **(B–E)**. Scale bars, 10 ms and 50 pA. *p < 0.05 by Mann–Whitney U test. **(F)** Summary plots of disparity index (square) and disparity ratio (circle) in wild-type (open squares and circles) and NL2-KO (blue squares and circles) mice at P5-P6, P7-P9, P10-P12, P13-P15, P16-P20, and P22-P35. Both disparity index and disparity ratio of NL2-KO mice were comparable to those of wild-type mice (Mann–Whitney test with FDR correction using the Benjamini-Hochberg procedure). The numbers of tested PCs are shown in parentheses below the graph. Data are expressed as mean ± SD.

To investigate at which stage of postnatal development the abnormality of synapse elimination takes place in NL2-KO mice, we followed the developmental course of CF innervation. At P5-P6, just before the onset of synapse elimination, the majority of PCs were innervated by three or more CFs in both strains of mice with no significant difference in the frequency distribution of PCs in terms of the number of CF-EPSC steps per PC (Figure 5A, *p* = 0.628, Mann–Whitney U test). At P7-P9, the frequency distributions of PCs markedly shifted to smaller numbers from those at P5-P6 in both strains of mice and there was no significant difference between the two (Figure 5B, *p* = 0.103, Mann–Whitney U test). After P10, the number of CFs in each PC progressively decreased, but the degree of reduction was significantly smaller in NL2-KO mice than in wild-type mice. Consequently, the frequency distribution of PCs exhibited a significant difference between the two strains of mice at P10-P12, P13-P15, and P16-P19 (Figures 5C–E, P10-P12: *p* = 0.002; P13–P15: *p* = 0.044; P16-P19: *p* = 0.045, Mann–Whitney U test). These results indicate that initial formation of CF synapses and elimination of redundant CF synapses from P5 to P9 are normal but CF synapse elimination after P10 is impaired in NL2-KO mice.

To test whether functional differentiation into single “strong” CF and the other “weak” CFs in each PC is affected in NL2-KO mice, we calculated the two parameters termed disparity index and disparity ratio, which represent the degree of the functional differentiation of CF synapses (Hashimoto et al., 2009a; Hashimoto and Kano, 2003) (Figure 5F). We found that both parameters were similar between NL2-KO mice and wild-type mice throughout postnatal development and in young adulthood (Disparity ratio: P5-6: *p* = 0.41; P7-9: *p* = 0.782; P10-12: *p* = 0.692; P13-15: *p* = 0.98; P16-20: *p* = 0.565; P22-35: *p* = 0.642; Disparity index: P5-6: *p* = 0.188; P7-9: *p* = 0.775; P10-12: *p* = 0.913; P13-15: *p* = 0.886; P16-20: *p* = 0.583; P22-35: *p* = 0.642; Mann–Whitney test with FDR correction using the Benjamini-Hochberg procedure; Figure 5F). Taken together, the results so far indicate that CF synapse elimination after P10 is specifically impaired in PCs of NL2-KO mice, despite normal functional differentiation into single “strong” and the other “weak” CFs, normal CF synapse elimination from P5 to P9, and normal dendritic translocation of a single “strong” CF.

### Lack of NL2 in PCs is responsible for the impaired CF synapse elimination in NL2-KO mice

**Figure 6:**
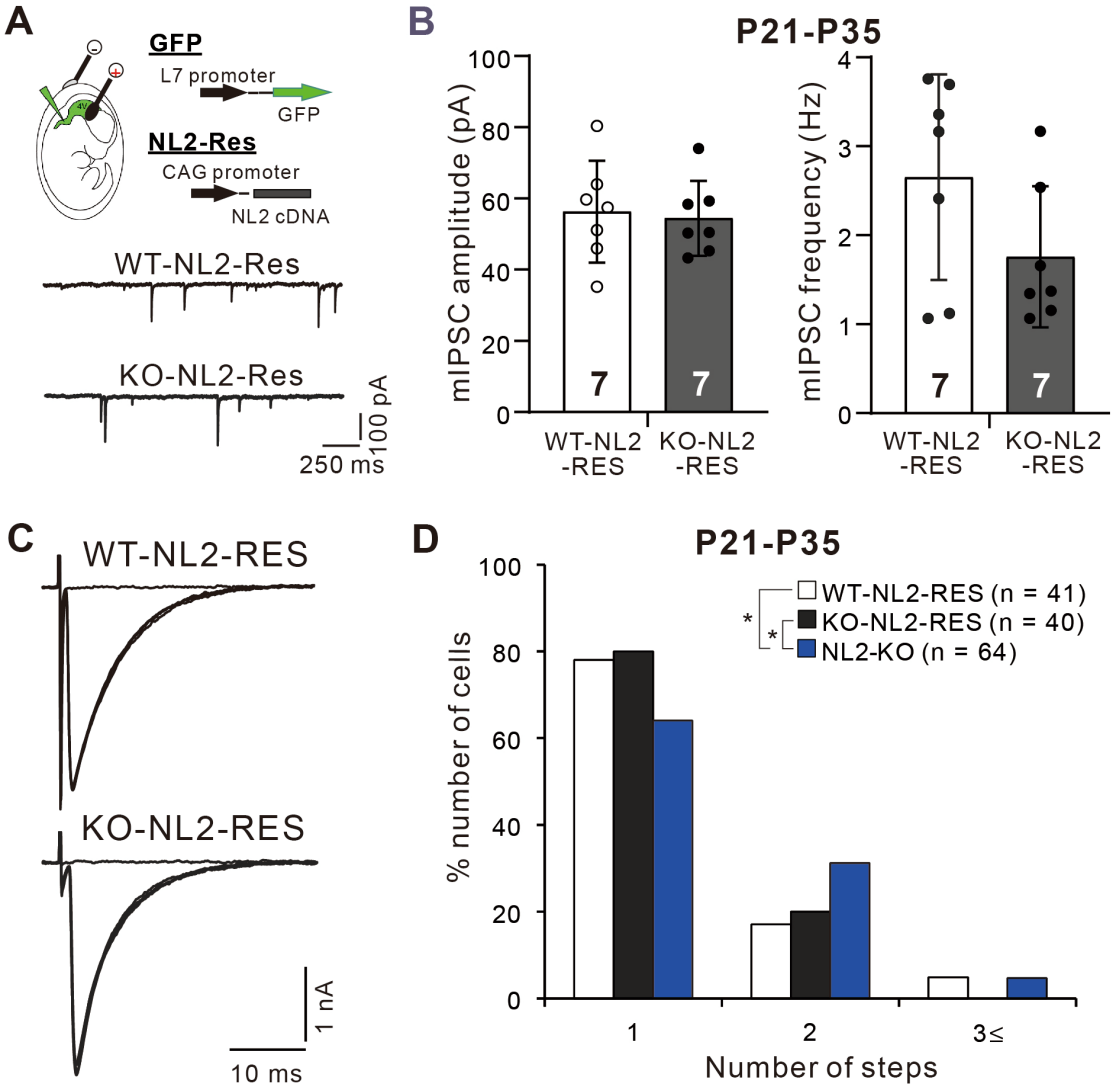
Rescue of the impaired inhibitory synaptic transmission and CF synapse elimination by expression of NL2 cDNA in PCs of NL2-KO mice. **(A)** Representative traces of mIPSC recorded from a PC with NL2 cDNA transfection in a wild-type (WT-NL2-Res) and an NL2-KO (KO-NL2-Res) mouse during P21-P35 in the presence of 1 μM TTX, 10 μM NBQX and 5 μM _R_-CPP. Scale bars, 250 ms and 100 pA. **(B)** Summary bar graphs and individual data distributions showing the amplitude and frequency of mIPSC in PCs transfected with NL2 cDNA in wild-type (open columns and open circles, *n* = 7) and NL2-KO (dark-columns and dark circles, *n* = 7) mice during P21-P35. There were no significant differences between the two groups by the Mann–Whitney U test. Data shown are means ± SD. The number of recorded cells is indicated within each column. **(C)** Representative traces of CF-EPSCs recorded in a PC with NL2 cDNA transfection in a wild-type and an NL2-KO mouse during P21-P35. Vh = −10 mV. Scale bars, 10 ms and 1 nA. **(D)** Frequency distributions showing the number of CFs innervating each PC in wild-type mice with PC-specific NL2 cDNA expression (open columns, *n* = 41) and NL2-KO PCs with (black columns, *n* = 40) or without (blue columns, *n* = 64) PC-specific NL2 cDNA expression during P21-P35. A significant difference was noted among the three groups (*p* = 0.041, Kruskal-Wallis test). *p < 0.05 by Dunn’s test for multiple comparisons.

Since NL2 is expressed widely in various cell types in the central nervous system (Patrizi et al., 2008; Varoqueaux et al., 2004), it is not clear whether the reduced GABAergic neurotransmission in PCs and impaired CF synapse elimination resulted from the lack of NL2 in PCs. To address this issue, we first examined whether PC-specific expression of NL2 in NL2-KO mice could rescue their cerebellar phenotypes. We introduced cDNA for NL2 together with that for EGFP into PCs of NL2-KO and wild-type mice at E11.5 or E12.5 by *in utero* electroporation. We used the same NL2 cDNA construct that was used to rescue the effects of microRNA-mediated NL2 knockdown (KD) in PCs (see Supplementary Figure S2). In this construct, seven nucleotides were mutated without changing the amino acid sequence in the microRNA targeted site so that the expression of NL2 was restored to normal level (Uesaka et al., 2012). We found that a subset of PCs expressed EGFP and no other cell types in the cerebellum did so. We made whole-cell recordings from EGFP-positive PCs that expressed NL2 and from EGFP-negative PCs in the same slices as the control. We found that after PC-specific expression of the NL2 cDNA, the mean amplitude (*p* = 0.62, Mann–Whitney U test) and frequency (*p* = 0.259, Mann–Whitney U test) of mIPSCs in PCs of NL2-KO mice were similar to those of wild-type mice (Figures 6A,B). Importantly, after PC-specific expression of NL2 cDNA, CF innervation patterns of EGFP-positive PCs during P21-P35 were identical between NL2-KO mice and wild-type mice, whereas CF innervation patterns were significantly different between EGFP-positive and EGFP-negative PCs in NL2-KO cerebellum (Kruskal-Wallis test: H(2) = 6.3886, *p* = 0.041; Dunn’s test for multiple comparisons: WT-NL2-RES vs NL2-KO: *p* = 0.0389; KO-NL2-RES vs NL2-KO: *p* = 0.0321; WT-NL2-RES vs KO-NL2-RES, *p* = 0.9321; Figures 6C,D). There were no significant changes in the basic electrophysiological properties of CF-EPSCs including the amplitude, the 10–90% rise time, the decay time constant, and the paired-pulse ratio (Supplementary Table S2). These results suggest that NL2 expression in PCs is sufficient to restore normal GABAergic transmission in PCs and normal CF synapse elimination during postnatal development.

**Figure 7:**
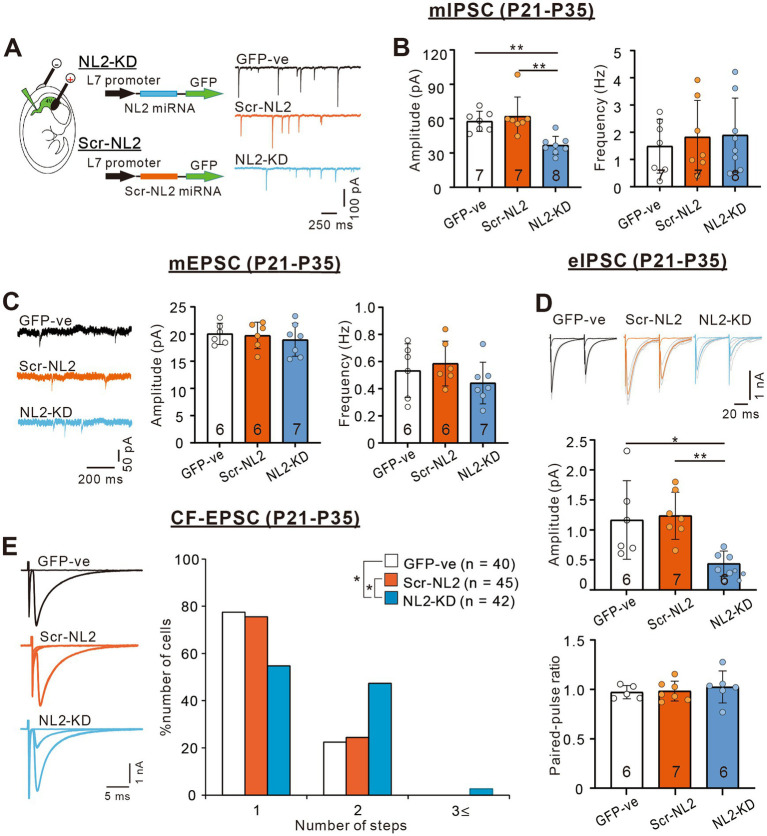
PC-specific NL2-KD caused the same phenotypes of reduced inhibitory transmission and impaired CF synapse elimination as in NL2-KO mice. **(A)** Representative traces of mIPSC recorded in a PC without transfection (untransfected GFP-ve, upper, black trace), with PC-specific scrambled NL2 expression (Scr-NL2, middle, orange trace) or with PC-specific NL2-KD (NL2-KD, lower, light blue trace) during P21-P35 in the presence of 1 μM TTX, 10 μM NBQX and 5 μM _R_-CPP. Vh = −70 mV. Scale bars, 250 ms and 100 pA. **(B)** Summary bar graphs and individual data distributions showing the mIPSC amplitudes (left) and frequency (right) for untransfected GFP-ve (open columns and open circles, *n* = 7), Scr-NL2 (orange columns and orange circles, *n* = 7), and NL2-KD (light blue columns and light blue circles, *n* = 8). Data are expressed as mean ± SD. The total number of cells recorded is indicated within each column. A significant difference was noted among the three groups for the amplitude (*p* < 0.001) but not for the frequency (*p* = 0.929) by the Kruskal-Wallis test. **p < 0.01 by Dunn’s test for multiple comparisons. **(C)** (Left) Representative traces of mEPSC recorded in an untransfected GFP-ve (Upper, black trace), Scr-NL2 (middle, orange trace), and NL2-KD (lower, light blue trace) PC during P21-P35 in the presence of 1 μM TTX and 0.1 mM Picotoxin. Vh = −70 mV. Scale bars, 200 ms and 50 pA. (Right) Summary bar graphs and individual data distributions showing the mEPSC amplitude and frequency of three groups of PCs. Data are expressed as mean ± SD. The total number of cells recorded is indicated within each column. No significant difference was detected among the three groups for either the amplitude (*p* = 0.786) or the frequency (*p* = 0.224) by the Kruskal-Wallis test. **(D)** (Upper) Representative traces of eIPSC recorded in an untransfected GFP-ve (left, black trace), Scr-NL2 (middle, orange trace) and NL2-KD (right, light blue trace) PC during P21-P35 in the presence of 1 μM TTX and 0.1 mM Picotoxin. Vh = −70 mV. Scale bars, 20 ms and 1 nA. (Lower) Summary bar graphs and individual data distributions showing the eEPSC amplitude and the paired-pulse ratio with 50 ms intervals of the three groups of PCs. Data are expressed as mean ± SD. The total number of cells recorded is indicated within each column. A significant difference was noted among the three groups for the amplitude (*p* = 0.002) but not for the frequency (*p* = 0.883) by the Kruskal-Wallis test. *p < 0.05 and **p < 0.01 by Dunn’s test for multiple comparisons. **(E)** (Left) Representative traces of CF-EPSCs from an untransfected GFP-ve control (upper, black trace), Scr-NL2 (middle, orange trace), and NL2-KD (lower, light blue trace) PC during P22–P35. Vh = −10 mV. Scale bars, 5 ms and 1 nA. (Right) Frequency distributions of the number of CFs innervating each PC for untransfected GFP-ve control (open columns, *n* = 40), Scr-NL2 (orange columns, *n* = 45), and NL2-KD (pure blue columns, *n* = 42) PCs during P22–P35. A significant difference was noted among the three groups (*p* = 0.0097, Kruskal-Wallis test). *p < 0.05 by Dunn’s test for multiple comparisons.

We then examined whether PC-specific knockdown (KD) of NL2 in wild-type mice causes reduced GABAergic transmission in PCs and impaired CF synapse elimination. We deleted NL2 specifically from PCs during postnatal development using the microRNA-mediated KD system (Uesaka et al., 2012; Uesaka et al., 2014). We introduced an NL2-KD vector together with EGFP cDNA into PCs under the control of a PC-specific L7 promoter by *in-utero* electroporation at E11.5–12.5. As a control, we similarly introduced scrambled NL2 microRNA into PCs (Figure 7A). We found that the amplitude of mIPSC in PCs with NL2-KD was significantly smaller than that of untransfected GFP-negative PCs or that of PCs expressing scrambled NL2 mRNA during P21-P35 (Kruskal-Wallis test: H(2) = 12.62, *p* < 0.001; Dunn’s test for multiple comparisons: GFP-ve vs. NL2-KD: *p* = 0.009; scrambled-NL2 vs. NL2-KD: *p* = 0.006), whereas no significant difference was found in the frequency of mIPSC among the three groups (Kruskal-Wallis test: H(2) = 0.1575, *p* = 0.929; Figure 7B). In contrast, the amplitude (Kruskal-Wallis test: H(2) = 0.5172, *p* = 0.786) and frequency (Kruskal-Wallis test: H(2) = 3.067, *p* = 0.224) of mEPSCs were similar among the three groups of PCs (Figure 7C). We also found that the amplitude of eIPSC in PCs with NL2-KD was significantly smaller than that of untransfected GFP-negative PCs or that of PCs expressing scrambled NL2 mRNA during P21 to P35 (Kruskal-Wallis test: H(2) = 10.18, *p* = 0.002; Dunn’s test for multiple comparisons: GFP-ve vs. NL2-KD: *p* = 0.041; scrambled-NL2 vs. NL2-KD: *p* = 0.008; Figure 7D). In contrast, no difference was found in the paired-pulse ratio of eIPSCs among the three groups of PCs (Kruskal-Wallis test: H(2) = 0.883, *p* = 0.663; Figure 7D). As for CF innervation, we found that a significantly higher percentage of PCs remain multiply innervated by CFs in PCs with NL2-KD during P22-P35 when compared with untransfected GFP-negative PCs or PCs expressing scrambled NL2 microRNA (Kruskal-Wallis test: (H(2) = 9.278, *p* = 0.0097; Dunn’s test for multiple comparisons: GFP-ve vs. NL2-KD: *p* = 0.0146; scrambled-NL2 vs. NL2-KD: *p* = 0.0468; GFP-ve vs scrambled-NL2: *p* = 0.724; Figures 7E). Co-expression of RNAi-resistant NL2 cDNA effectively rescued the impaired CF synapse elimination by NL2-KD in PCs (Supplementary Figure S2). There were no significant changes induced by NL2-KD in PCs in the basic electrophysiological properties of CF-EPSCs including the amplitude, the 10–90% rise time, the decay time constant, and the paired-pulse ratio (Supplementary Table S3). These results indicate that NL2 in PCs is required for normal GABAergic transmission in PCs and normal CF synapse elimination during postnatal development.

### Enhanced calcium transients induced by weaker CF inputs in PCs with NL2 deletion

**Figure 8:**
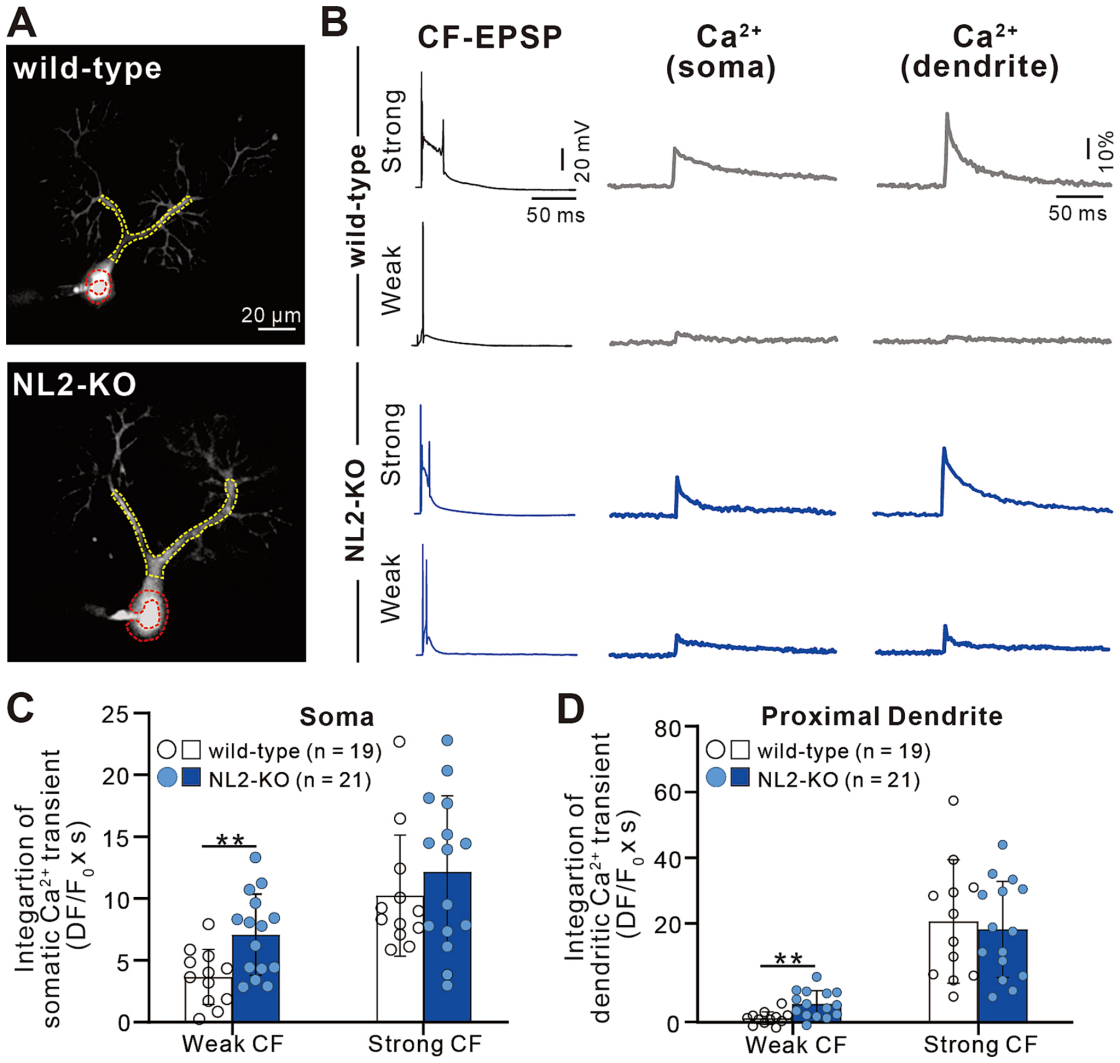
Enhanced calcium transients induced by activating a weak CF in PCs of NL2-KO mice. **(A)** Representative PC images of a wild-type and an NL2-KO mouse. Areas indicated by red and yellow dotted lines represent ROIs for somatic and dendritic calcium transients, respectively. Scale bar, 20 μm. **(B)** CF-EPSPs and calcium transients recorded in the soma and dendrite of a multiply innervated PC of a wild-type and an NL2-KO mouse in response to stimulation of a weaker or the strongest CF. Scale bars, 50 ms and 20 mV for EPSPs, 50 ms and 10% for calcium transients. **(C,D)** Summary bar graphs and individual data distributions showing average magnitudes of calcium transients from the PC soma **(C)** and dendrites **(D)** by stimulating a weaker or the strongest CF. Calcium transients for 1.5 s from the onset of CF stimulation were integrated. Data are expressed as mean ± SD. *p < 0.05 and **p < 0.01 by the Mann–Whitney test with FDR correction using the Benjamini-Hochberg procedure.

The results so far indicate that lack of NL2 in PCs results in persistent multiple CF innervation due to impaired CF synapse elimination after P10. We hypothesize that reduced GABAergic inhibition in PCs with NL2 deletion was the main cause of impaired CF synapse elimination. CF inputs depolarize PCs and induce calcium transients mediated mainly by P/Q-type voltage-gated calcium channels (P/Q-VDCC) *in vivo*. Our previous studies indicate that calcium transients of PCs by the activation of P/Q-VDCC are crucial for CF synapse elimination (Hashimoto et al., 2011; Kawata et al., 2014; Mikuni et al., 2013). We therefore examined whether CF-induced calcium transients were altered in NL2-lacking PCs with reduced GABAergic inhibition. We made simultaneous whole-cell recordings under current-clamp mode and intracellular calcium measurements from PCs at P10-P12 in normal external solution without GABAergic blockers. We selected PCs that were multiply innervated by a single “strong” CF (CF-multi-S) and one or two “weak” CFs (CF-multi-W) (Figures 8A,B). Activation of CF-multi-S induced characteristic complex spikes and large calcium transients in the soma and proximal dendrites of PCs in both wild-type and NL2-KO mice (Figure 8B). We found no significant differences between the two genotypes in somatic (Figure 8C; *p* = 0.459, Mann–Whitney test with FDR correction using the Benjamini-Hochberg procedure) and proximal dendritic (Figure 8D; *p* = 0.905, Mann–Whitney test with FDR correction using the Benjamini-Hochberg procedure) calcium transients. In contrast, stimulation of CF-multi-W induced relatively small EPSPs with one or two spikes and relatively small calcium transients when compared with stimulation of CF-multi-S in wild-type mice. Importantly, stimulation of CF-multi-W in NL2-KO mice induced significantly larger calcium transients in both somata (Figure 8C; *p* = 0.0098, Mann–Whitney test with FDR correction using the Benjamini-Hochberg procedure) and proximal dendrites (Figure 8D; *p* = 0.00176, Mann–Whitney test with FDR correction using the Benjamini-Hochberg procedure) than in wild-type mice.

**Figure 9:**
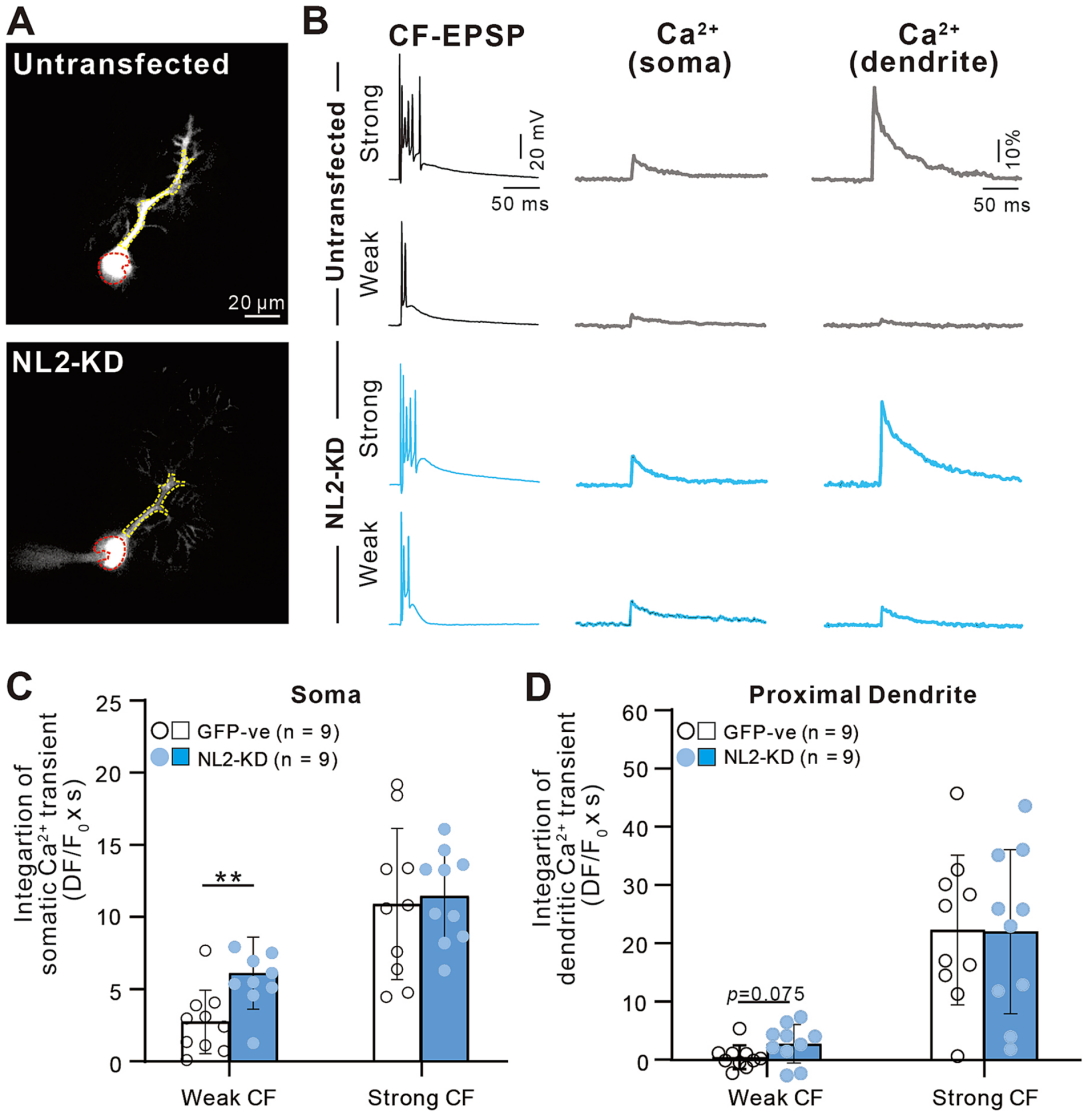
Enhanced calcium transients induced by activating a weak CF in PCs with of NL2-KD. **(A)** Representative PC images of an untransfected GFP-ve (upper) and a PC-specific NL2- KD (lower) mouse. Scale bar, 20 μm. **(B)** CF-EPSPs and calcium transients recorded in the soma and dendrite of a multiply innervated PC of an untransfected GFP-ve (upper) and a PC-specific NL2- KD (lower) mouse in response to stimulation of a weaker or the strongest CF. Scale bars, 50 ms and 20 mV for EPSPs, 50 ms and 10% for calcium transients. **(C,D)** Summary bar graphs and individual data distributions showing average magnitudes of calcium transients from the PC soma **(C)** and dendrites **(D)** by stimulating a weaker or the strongest CF. Calcium transients for 1.5 s from the onset of CF stimulation were integrated. Data are expressed as mean ± SD. *p < 0.05 and **p < 0.01 by the Mann–Whitney test with FDR correction using the Benjamini-Hochberg procedure.

We also investigated whether a similar enhancement of calcium transients induced by weaker CF inputs was seen in PCs with NL2-KD (Figure 9). Activation of CF-multi-S induced characteristic complex spikes and large calcium transients in both somata and proximal dendrites with no significant differences between control and NL2-KD PCs (Figures 9A–D, Somata: *p* = 0.8; Proximal dendrite: *p* = 0.912, Mann–Whitney U test). In contrast, stimulation of CF-multi-W induced larger calcium transients in both somata and proximal dendrites in NL2-KD PCs than in control PCs (Figures 9A–D, Somata: *p* = 0.005; Proximal dendrites: *p* = 0.075, Mann–Whitney U test). Taken together, these results indicate that deletion of NL2 in PCs attenuates GABAergic inhibition in PCs, which allows weaker CFs to generate relatively large calcium transients in the soma and proximal dendrites. Large calcium transients may enhance the activation of calcium-dependent reinforcement signals for synapses and therefore may permit weak CF synapses to survive on the PC soma.

**Figure 10:**
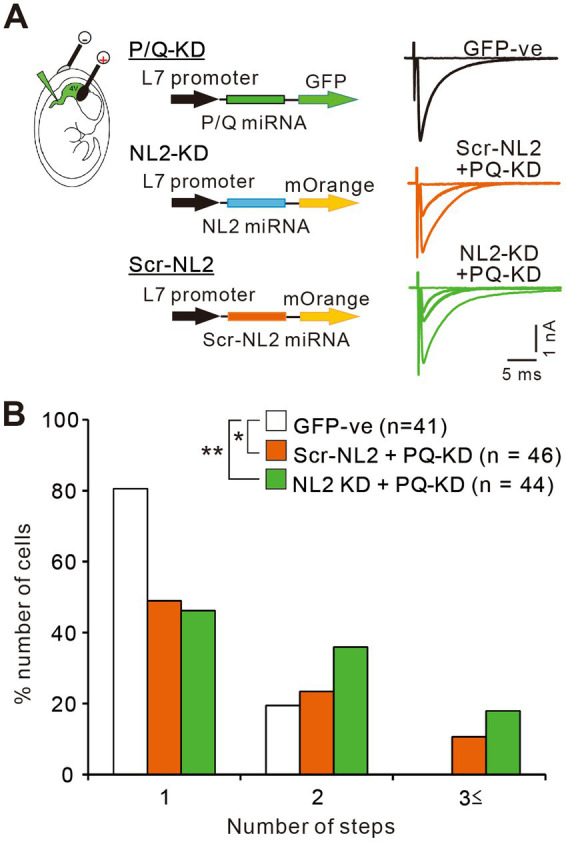
P/Q-VDCC is involved in the persistent multiple CF innervations in NL2-KD PCs. **(A)** Representative traces of CF-EPSCs from a PC without transfection (GFP −ve, black trace), a PC with Scrambled NL2-KD + P/Q-KD (Scr-NL2 + PQ-KD, middle, orange trace), and a PC with NL2-KD + P/Q-KD (NL2-KD + PQ-KD, lower, green trace) during P22–P35 in the presence of 0.1 mM picotoxin. Vh = −10 mV. Scale bars, 5 ms and 0.5 nA. **(B)** Frequency distributions of the number of CFs innervating each PC for untransfected GFP-ve control PCs (open columns, *n* = 41), PCs with Scr-NL2 + P/Q-KD (orange columns, *n* = 46), and PCs with NL2-KD + P/Q-KD (green columns, *n* = 44) during P22-P35. A significant difference was noted among the three groups (*p* = 0.0027, Kruskal-Wallis test). *p < 0.05 and **p < 0.01 by Dunn’s test for multiple comparisons.

Previous studies indicate that calcium transients induced by CF inputs are mediated mainly by P/Q-VDCCs (Hashimoto et al., 2011; Otsu et al., 2014). It is therefore possible that enhanced calcium influx through P/Q-VDCCs might facilitate the survival of weak CF inputs in PCs lacking NL2. To check whether P/Q-VDCC is involved in the persistent multiple CF innervation in PCs lacking NL2, we performed double KD of NL2 and P/Q-VDCC in PCs and compared the effect with that of single P/Q-VDCC KD (Figure 10). As reported previously (Kawata et al., 2014; Mikuni et al., 2013; Uesaka et al., 2014), the knockdown of P/Q-VDCC in PCs caused a significant impairment of CF synapse elimination. In PCs expressing microRNA against P/Q-VDCC together with scrambled NL2 microRNA (Scr-NL2 + PQ KD), about 50% of PCs remained multiply innervated by CFs and their frequency distribution in terms of the number of CFs innervating each PC was significantly different from that of untransfected GFP-negative PCs (Figures 10A,B). We then performed double KD of NL2 and P/Q-VDCC in PCs and found that the effect of KD was similar to that of single KD of P/Q-VDCC (Figures 10A,B). Frequency distribution histograms were almost identical between PCs expressing microRNA against NL2 together with that against P/Q-VDCC (NL2-KD + PQ-KD) and PCs with single KD of P/Q-VDCC (Scr-NL2 + PQ-KD) (Kruskal-Wallis test: (H(2) = 11.83, *p* = 0.0027; Dunn’s test for multiple comparisons: GFP-ve vs. Scr-NL2 + P/Q-VDCC, *p* = 0.0424; GFP-ve vs. NL2-KD + P/Q-VDCC, *p* = 0.0025; NL2-KD+P/Q-VDCC vs Scr-NL2 + P/Q-VDCC, *p* > 0.9999; Figures 10A,B). No significant differences that appeared functionally important were found among the three groups of PCs in the basic electrophysiological properties of CF-EPSCs including the amplitude, the 10-90% rise time, the decay time constant, and the extent of the paired-pulse ratio (Supplementary Table S4). The occlusion of the effect of NL2-KD by simultaneous P/Q-VDCC KD suggests that calcium influx through P/Q-VDCC into PCs is involved in the persistent multiple CF innervations in NL2-deleted PCs.

## Discussion

In the present study, we demonstrated that deletion of NL2 in cerebellar PCs caused attenuation of GABAergic inhibitory transmission to PCs and persistent multiple CF innervation of PCs. GABAergic inhibition to PCs was attenuated in NL2-KO mice particularly from P10 to P12 when massive formation of inhibitory synapses occurs from molecular layer interneurons to PCs (Yamanaka et al., 2004). Elimination of redundant CF synapses was impaired after P10. In contrast, selective strengthening of a single CF in each PC and dendritic translocation of the strengthened CF were normal. While calcium transients induced by activation of the strongest CF in the soma and proximal dendrites were normal in PCs with NL2 deletion at P10-P12, those caused by activation of the weaker CF were larger than in control PCs. The impaired CF synapse elimination in PCs with NL2-KD was occluded by simultaneous KD of P/Q-VDCC in PCs, suggesting that P/Q-VDCC mediates the effect of NL2 deletion on CF synapse elimination.

Our results of reduced GABAergic inhibition in NL2-deficient PCs during postnatal development are complementary to the previous report by Zhang et al. (2015) that inhibitory synaptic transmission was attenuated in PCs of mature PC-specific NL2 conditional KO mice at P21-P25 or older (Zhang et al., 2015). However, they did not examine CF synapse elimination during postnatal development in their PC-specific NL2 conditional KO mice (Zhang et al., 2015). Moreover, while Zhang et al. (2015) reported an increase in the amplitude of CF-EPSCs and a decrease in the mIPSC frequency in mature PC-specific NL2 conditional KO mice (Zhang et al., 2015), we did not observe such changes in the present study. The reasons for the discrepancies are unclear but might be attributed to differences in experimental conditions and methods of NL2 deletion. Despite some discrepancies in the results, both studies consistently show the role of NL2 in maintaining inhibitory synaptic function in PCs.

The present study advanced our previous preliminary report that RNAi-mediated NL2-KD in PCs of olivo-cerebellar coculture preparations increased the number of CFs innervating individual PCs (Uesaka et al., 2012). We elucidated how the lack of NL2 in PCs affects inhibitory synaptic function, identified the stage of postnatal development when NL2 is required for CF synapse elimination, and showed how weaker CF inputs induce larger calcium transients in NL2-deficient PCs than in normal PCs. The roles of NL2 in synapse formation and function are well-established, particularly through its interactions with gephyrin and collybistin to stabilize GABA_A_ receptor clusters at inhibitory synapses (Poulopoulos et al., 2009; Varoqueaux et al., 2004). In NL2-deficient PCs, disruption of these interactions is thought to cause the attenuation of GABAergic inhibition without affecting excitatory synaptic function, leading to elevation of E/I balance and enhanced calcium signaling in response to the stimulation of weaker CFs.

We have reported previously that heterozygous GAD67-KO mice exhibit reduced GABAergic inhibition from basket cells to PCs at P10-P12, impaired CF synapse elimination from P10, and increased calcium transients induced by activating a weaker CF in the soma despite normal calcium transients induced by activation of the strongest CF (Nakayama et al., 2012). The phenotypes of heterozygous GAD67-KO mice are essentially the same as those of NL2-KO mice and mice with NL2-KD in PCs. Since the elimination of redundant CFs proceeds in two distinct phases, the early phase from around P7 to around P11 and the late phase from around P12 to around P17, these results indicate that part of the early phase and the late phase of CF elimination are dependent on GABAergic inhibition and require NL2 in PCs. Nakayama et al. (2018) reported that deletion of microglia in the cerebellum during postnatal development impaired CF synapse elimination from around P10 due to the impaired maturation of GABAergic inhibition from molecular layer interneurons to PCs. Moreover, mice deficient in the receptor tyrosine kinase TrkB showed a moderate delay in the maturation of GABAergic synapses and abnormal persistent multiple CF innervation in PCs (Bosman et al., 2006). Since the excitatory synaptic transmission in PCs was not affected in mice with NL2 deletion in PCs, heterozygous GAD67-KO mice (Nakayama et al., 2012), mice with microglia deletion in the developing cerebellum (Nakayama et al., 2018), and TrkB-KO mice (Bosman et al., 2006), the E/I balance of synaptic transmission is thought to be elevated in these mouse models. These results suggest that proper maturation of GABAergic inhibition to PCs is a prerequisite for accomplishing the CF to PC mono-innervation pattern during postnatal development.

Our results support a causal link between NL2 reduction and excess calcium signaling in PCs. First, we show that the deletion of NL2 in cerebellar PCs reduced GABAergic inhibition of PCs without affecting excitatory synaptic function (Figures 1,7), leading to the elevation of E/I balance. Second, our calcium imaging from PCs revealed significantly elevated calcium transients in response to the stimulation of weaker CFs in NL2-deficient PCs than in control PCs (Figures 8,9). The reduced inhibition is thought to allow weaker CF inputs to induce larger excitatory postsynaptic potentials and stronger activation of postsynaptic P/Q-VDCCs, resulting in enhanced calcium transients in NL2-deleted PCs compared to control PCs. Third, the reduced GABAergic inhibition in PCs by a cause other than NL2 deletion, i.e., heterozygous GAD67 deletion (Nakayama et al., 2012), has been reported to result in elevated calcium signaling in PCs in response to the stimulation of weaker CFs. This indicates that reduced GABAergic inhibition in PCs, irrespective of its cause, leads to enhanced calcium signaling in response to weaker CF inputs. Fourth, when we introduced an NL2 KD construct in combination with a P/Q-VDCC KD construct, the effect of NL2 KD on CF synapse elimination was occluded (Figure 10) implying that the effect of NL2 deletion on CF synapse elimination is largely dependent on calcium influx to PCs through P/Q-VDCC. These lines of evidence indicate that the deletion of NL2 and excess calcium signaling in PCs are not independent phenomena but causally linked.

How does attenuated inhibition to PCs result in persistent multiple CF innervation? In both heterozygous GAD67-KO mice and NL2-KO mice, activation of a weaker CF in PCs innervated by single strong CFs and weaker CFs induced larger calcium transients than in wild-type mice at P10-P12, suggesting that GABAergic inhibition influences calcium-dependent mechanisms of CF elimination. Calcium transients induced by CF activity are thought to be mediated mainly by P/Q-VDCC (Hashimoto et al., 2011; Otsu et al., 2014). Our previous studies show that global and PC-selective P/Q-VDCC-KO mice are impaired in the selective strengthening of a single CF from around P3 to P7, the dendritic translocation of the single “winner” CF from around P9, the early phase of CF elimination from around P7 to P11 (Hashimoto et al., 2011; Miyazaki et al., 2004) and the late phase of CF elimination from around P12 to P17 (Kawata et al., 2014; Mikuni et al., 2013). Our present results showed that the effect of NL2-KD in PCs on CF innervation was occluded when P/Q-VDCC was knocked down simultaneously in PCs. These results suggest that NL2 deletion in PCs perturbs calcium-dependent CF elimination mediated by P/Q-VDCC from P10.

On the other hand, we reported previously that mice with the arginine to cysteine substitution at the 451st amino acid residue (R451C) of neuroligin-3 (NL3) exhibited an enhancement of GABAergic inhibition to PCs and a transient impairment of CF synapse elimination from P10 to P15 (Lai et al., 2021). Calcium transients in the PC soma elicited by stimulating both strong and weak CFs were significantly smaller in NL3-R451C mutant mice than in wild-type mice. Whereas most PCs became mono-innervated after P16 in NL3-R451C mutant mice as in wild-type mice, the amplitude of EPSCs for weaker CFs in PCs with multiple CF innervation was significantly larger in NL3-R451C mutant mice than in wild-type mice (Lai et al., 2021). These results indicate that the reduction of GABAergic inhibition in PCs (such as NL2-KO mice and heterozygous GAD67-KO mice) and the enhancement of PC inhibition (NL3-R451C mutant mice) result in apparently similar phenotypes of impaired CF synapse elimination. How do the changes in the strength of GABAergic inhibition in the opposite directions cause apparently similar phenotypes?

It is thought that, during developmental synapse refinement, stronger synaptic inputs generate “punishment signals” that trigger “elimination signals” at weaker synapses to depress them but spare the stronger synapses themselves (Lichtman and Colman, 2000; Nagappan-Chettiar et al., 2023). By analogy, we assume that the strongest CF inputs may produce “punishment signals” that depress the weaker CF inputs by generating “elimination signals.” The hypothetical punishment signals are assumed to require large calcium transients in PCs for their production hence, weaker CF inputs are considered unable to generate them. Moreover, the strongest CF inputs that generate large calcium transients should be protected from such punishment signals. We also hypothesize the presence of “survival signals” or “stabilization signals” that are produced by calcium transients in PCs and are necessary for the maintenance of CF inputs. In wild-type mice, most PCs become mono-innervated by single strong CFs during the two phases of CF elimination presumably because weaker CFs are eliminated by elimination signals triggered by punishment signals derived from the strongest CFs which may overcome the survival signals derived from the weaker CFs. In NL2-KO mice and mice with PC-specific NL2-KD, the weaker CFs generate relatively large calcium transients in PCs and may produce survival signals sufficient to protect the weaker CFs from the elimination signals. In NL3-R451C mutant mice, activation of the strongest CF induced smaller calcium transients in the soma than in wild-type mice (Lai et al., 2021), which may not produce sufficient punishment signals enough to generate elimination signals to eliminate weaker CFs. Consequently, CF synapse elimination may be transiently impaired from P10 to P15 in NL3-R451C mutant mice (Lai et al., 2021). However, subsets of the weaker CFs in NL3-R451C mutant mice might not be able to produce sufficient survival signals hence they may be eliminated eventually. In contrast, the weaker CF inputs of NL3-R451C mutant mice that survived into the juvenile stage may be stronger than those of wild-type mice presumably because of the reduced production of the hypothetical punishment signals from the strongest CF inputs and insufficient production of elimination signals.

CF synapse elimination in the developing cerebellum is critically dependent on the activity of PCs (Lorenzetto et al., 2009). The P/Q-VDCC and the type 1 metabotropic glutamate receptor (mGluR1) are shown to trigger two canonical activity-dependent pathways in PCs for the multiple processes of CF synapse elimination (Kano and Watanabe, 2019; Kano et al., 2018). The mGluR1 and its downstream signaling pathway in PCs involving Gαq (Offermanns et al., 1997), phospholipase C β3 and β4 (PLCβ3, PLCβ4) (Kano et al., 1998; Rai et al., 2021), and protein kinase Cγ (PKCγ) (Kano et al., 1995) are crucial for the late phase of CF elimination. The mGluR1 is shown to be activated at PF-PC synapses by the activity along the mossy fiber-granule cell-PF circuit and promotes the elimination of redundant weaker CF synapses (Nakayama et al., 2024). The semaphorin 7A-Plexin C1/Integrin B1 (Uesaka et al., 2014) and BDNF–TrkB (Choo et al., 2017) are shown to function as elimination signals along the mGluR1 signaling pathway. In contrast, the immediate early gene *Arc* is reported to be an elimination signal along the P/Q-VDCC signaling pathway for the late phase of CF elimination (Mikuni et al., 2013). On the other hand, the semaphorin 3A-Plexin A4 (Uesaka et al., 2014), progranulin-Sort 1 (Uesaka et al., 2018), and C1q-like molecule 1 (C1ql1)- cell adhesion G-protein-coupled receptor 3 (Bai3) (Kakegawa et al., 2015) are reported to function as stabilization factors. However, whether these molecules are functional at the downstream of P/Q-VDCC or mGluR1 is not known. Moreover, the nature of punishment signals is not known. In mitral cells of the developing olfactory bulb, the elimination of dendritic branches is mediated by RhoA generated at synapses on the dendritic branch that will survive, indicating that RhoA functions as a punishment signal (Fujimoto et al., 2023). Since RhoA is shown to be activated by calcium entry through NMDA receptors on the surviving dendritic blanch of olfactory mitral cells (Fujimoto et al., 2023), analogous mechanisms that are triggered by P/Q-VDCC-mediated calcium entry may exist at winning CF synapses on PC dendrites. Further investigation is needed to identify “punishment signals,” “elimination signals,” and “stabilization signals” and elucidate how these signals work at multiple stages of CF synapse elimination and maturation during postnatal cerebellar development.

## Data Availability

The raw data supporting the conclusions of this article will be made available by the authors, without undue reservation.

## Footnotes

^1^ http://rsb.info.nih.gov/ij/
