# Supplementary material for: Reduced GABAergic inhibition and impaired synapse elimination by neuroligin-2 deletion from Purkinje cells of the developing cerebellum
Source: Front Neural Circuits. 2025 Mar 14;19:1530141. doi: 10.3389/fncir.2025.1530141 (PMC11949940; doi:10.3389/fncir.2025.1530141)
Supplement: Supplementary file 1 [file Data_Sheet_1.pdf]

Supplementary Material:

Reduced GABAergic inhibition and impaired synapse elimination by neuroligin-2 deletion from Purkinje cells of the developing cerebellum

1 Supplementary Figures and Tables

1.1 Supplementary Figures

Supplementary Figure 1

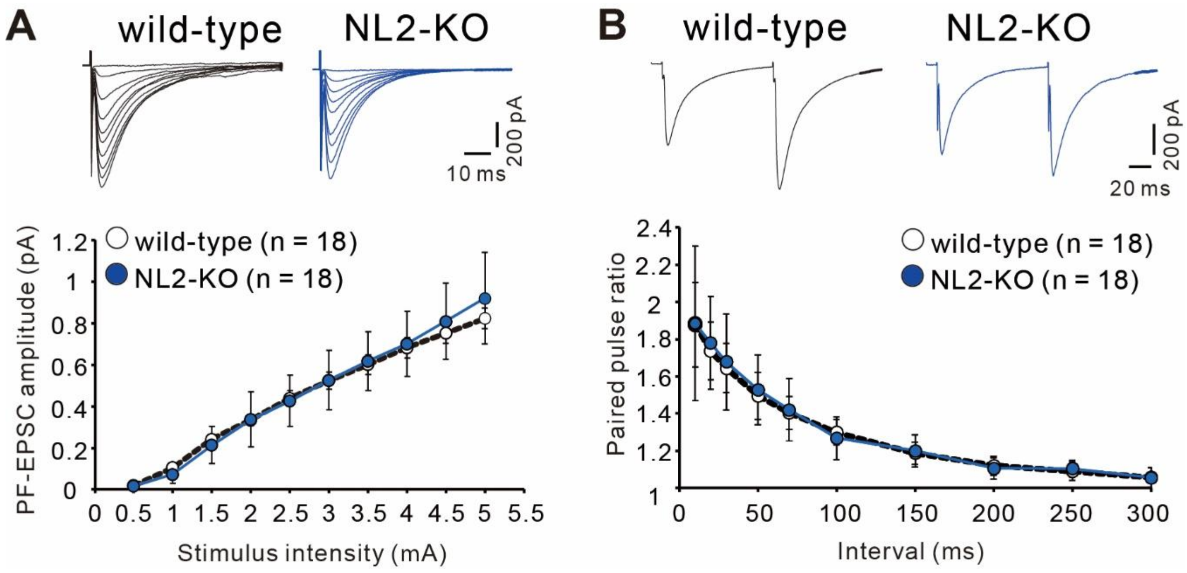

Supplementary Figure 1. Unaltered PF-EPSCs in NL2-KO mice.

(A) Input-output relationship of PF-EPSCs. Representative traces of PF-EPSCs (upper) and summary plots (lower) at different stimulus intensities in wild-type (black traces and open circles, n = 18) and NL2-KO (blue traces and filled circles, n = 18) PCs during P22-P35. Vh = -70 mV. Scale bars, 10 ms and 200 pA. Data are expressed as mean ± SD. Note that there were no significant differences between wild-type and NL2-KO mice (0.5 μA: p = 0.973; 1 μA: p = 0.322; 1.5 μA: p = 0.394; 2 μA: p = n.s.; 2.5 μA: p = 0.708; 3 μA: p = 0.61; 3.5 μA: p = 0.892; 4 μA: p = 0.786; 4.5 μA: p = 0.973; 5 μA: p = 0.973; Mann-Whitney test with FDR correction using the Benjamini-Hochberg procedure).

(B) Paired-pulse ratio of PF-EPSCs. Representative traces of PF-EPSCs (upper) and summary plots (lower) at different inter-stimulus intervals in wild-type (black traces and open circles, n = 18) and NL2-KO (blue traces and filled circles, n = 18) PCs during P22-P35. Vh = -70 mV. Scale bars, 200 ms and 200 pA. Data are expressed as mean ± SD. There were no significant differences between the genotypes (10 ms: p = 0.521; 20 ms: p = 0.913; 30 ms: p = 0.725; 50 ms: p = 0.791; 70 ms: p = 0.339; 100 ms: p = 0.389; 150 ms: p = 0.526; 200 ms: p = 0.406; 250 ms: p = 0.223; 300 ms: p = 0.913; Mann-Whitney test with FDR correction using the Benjamini-Hochberg procedure).

Supplementary Figure 2

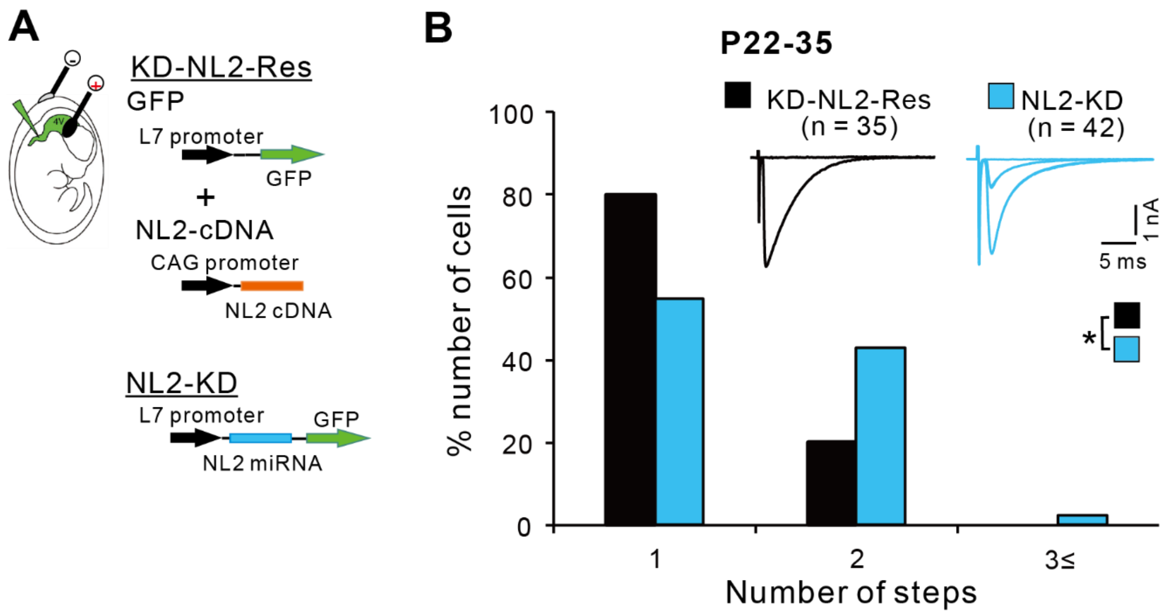

Supplementary Figure 2. Rescue of the impaired CF synapse elimination by expression of RNAi-resistant form of NL2 cDNA in PCs with NL2-KD.

(A) Schema showing the transfection of microRNA against NL2 (NL2-KD) or a combination of EGFP cDNA, RNAi-resistant NL2 cDNA, and microRNA against NL2 (KD-NL2-Res) into PCs by *in utero* electroporation.

(B) Representative traces of CF-EPSCs (inset) and frequency distribution histograms for the number of CF-EPSC steps in individual PCs with co-transfection of NL2-KD, EGFP cDNA, and NL2 cDNA (black columns, n = 35) and PCs with NL2-KD (light blue columns, n = 42) during P22-P35. *p = 0.019, Mann-Whitney U test.

1.2 Supplementary Tables

Supplementary Table 1. Electrophysiological Parameters of CF-EPSCs in PCs of wild-type and NL2-KO mice (P22–P35).

	CF-group	Number of cells	Amplitude (nA)	10-90% Rise time (ms)	Decay time constant (ms)	CF-PPR (50 ms)
WT	CF-mono	33	2.73 ± 0.86	0.44 ± 0.07	6.76 ± 1.05	0.72 ± 0.05
	CF-multi-S	10	2.41 ± 0.76	0.46 ± 0.59	6.89 ± 1.77	0.76 ± 0.41
	CF-multi-W	8	0.58 ± 0.39	0.39 ± 0.03	3.18 ± 2.26	0.71 ± 0.08
NL2-KO	CF-mono	34	2.85 ± 1.01	0.45 ± 0.03	6.0 ± 1.09**	0.74 ± 0.06
	CF-multi-S	16	3.17 ± 1.29	0.43 ± 0.07	5.36 ± 0.93*	0.76 ± 0.03
	CF-multi-W	16	0.80 ± 0.64	0.46 ± 0.14	4.80 ± 2.48	0.72 ± 0.04

All data are expressed as mean ± SD. CF-EPSCs are divided into three groups, namely CF-EPSCs from mono-innervated PCs (CF-mono), the largest CF-EPSCs in multiply innervated PCs (CF-multi-S), and the other smaller CF-EPSCs in multiply innervated PC (CF-multi-W). Amplitudes of all CF-EPSCs were measured at Vh = -10 mV. The decay time constant was obtained by fitting the EPSC decay with a single exponential. The paired-pulse ratio (PPR) was defined as the percent of the second EPSC amplitude relative to the first one with 50 ms inter-stimulus interval.

There were no significant differences between wild-type and NL2-KO mice in the amplitude, 10%–90% rise time, and paired-pulse ratio of EPSCs for CF-mono, CF-multi-S, and CF-multi-W (amplitude: CF-mono: $p = 0.800$; CF-multi-S: $p = 0.799$; CF-multi-W: $p = 0.642$; 10-90% rise time: CF-mono: $p = 0.432$; CF-multi-S: $p = 0.722$; CF-multi-W: $p = 0.53$; paired-pulse ratio: CF-mono: $p = 0.197$; CF-multi-S: $p = 0.738$; CF-multi-W: $p = 0.833$; Mann-Whitney U test). The decay time constant of EPSCs for CF- mono and that for CF-multi-S (CF-mono: $p = 0.007$; CF-multi-S: $p = 0.029$) but not that for CF-multi-W (CF-multi-W: $p = 0.628$) were slightly shorter in NL2-KO PCs than in wild-type PCs (Mann-Whitney U test).

*$p < 0.05$ and **$p < 0.01$ by Mann Whitney U test.

Supplementary Table 2. Electrophysiological Parameters of CF-EPSCs in PCs of wild-type and NL2-KO mice transfected with NL2 cDNA (P22–P35).

	CF-group	Number of cells	Amplitude (nA)	10-90% Rise time (ms)	Decay time constant (ms)	CF-PPR (50 ms)
WT-NL2-RES	CF-mono	25	2.87 ± 0.99	0.54 ± 0.13	5.26 ± 1.09	0.70 ± 0.08
	CF-multi-S	9	2.49 ± 1.5	0.47 ± 0.02	6.17 ± 1.13	0.78 ± 0.05
	CF-multi-W	9	0.37 ± 0.34	0.57 ± 0.14	4.17 ± 1.59	0.77 ± 0.04
KO-NL2-RES	CF-mono	25	2.33 ± 0.69	0.53 ± 0.1	5.54 ± 1.59	0.73 ± 0.06
	CF-multi-S	8	2.31 ± 1.9	0.61 ± 0.19	4.79 ± 1.41	0.77 ± 0.11
	CF-multi-W	8	0.67 ± 0.54	0.47 ± 0.21	4.58 ± 0.89	0.78 ± 0.12

Data were obtained, analyzed, and expressed as shown in Table S1.

There were no significant differences between wild-type and NL2-KO PCs in the basic electrophysiological parameters of the three types of CF-EPSCs including the amplitude (CF-mono: p = 0.051; CF-multi-S: p = 0.606; CF-multi-W: p = 0.277; Mann-Whitney U test), the 10%-90% rise time (CF-mono: p = 0.927; CF-multi-S: p = 0.133; CF-multi-W: p = 0.303; Mann Whitney U-test), the decay time constant (CF-mono: p = 0.337; CF-multi-S: p = 0.267; CF-multi-W: p = 0.94; Mann-Whitney U test), and the extent of the paired-pulse ratio (CF-mono: p = 0.255; CF-multi-S: p = 0.8; CF-multi-W: p = 0.973; Mann-Whitney U test).

Supplementary Table 3. Electrophysiological Parameters of CF-EPSCs in uninfected PCs, PCs with NL2-KD, and PCs expressing scrambled NL2 (P22–35).

	CF-group	Number of cells	Amplitude (nA)	10-90% Rise time (ms)	Decay time constant (ms)	CF-PPR (50 ms)
Uninfected	CF-mono	29	2.28 ± 0.07	0.52 ± 0.09	6.4 ± 1.84	0.77 ± 0.05
	CF-multi-S	10	2.28 ± 0.62	0.51 ± 0.08	5.12 ± 0.62	0.72 ± 0.03
	CF-multi-W	10	0.66 ± 0.48	0.58 ± 0.19	4.38 ± 1.62	0.69 ± 0.04
NL2-KD	CF-mono	25	2.56 ± 0.96	0.76 ± 0.87	5.78 ± 1.07	0.76 ± 0.07
	CF-multi-S	17	1.98 ± 0.17	0.51 ± 0.08	5.16 ± 1.39	0.65 ± 0.1
	CF-multi-W	17	0.52 ± 0.32	0.49 ± 0.12	3.37 ± 0.05	0.69 ± 0.15
Scr-NL2	CF-mono	28	2.49 ± 0.04	0.55 ± 0.09	5.79 ± 1.65	0.75 ± 0.06
	CF-multi-S	11	1.98 ± 1.06	0.47 ± 0.09	4.93 ± 1.4	0.73 ± 0.09
	CF-multi-W	11	0.69 ± 0.5	0.49 ± 0.16	4.26 ± 1.63	0.71 ± 0.11

Data were obtained and expressed as shown in Tables S1 and S2.

There were no significant differences among the three groups of PCs in the basic electrophysiological properties of the three types of CF-EPSCs including the amplitude (CF-mono: $H(2) = 1.222$, $p = 0.543$; CF-multi-S: $H(2) = 4.017$, $p = 0.134$; CF-multi-W: $H(2) = 0.639$, $p = 0.726$; Kruskal-Wallis test), the 10%-90% rise time (CF-mono: $H(2) = 5.556$, $p = 0.062$; CF-multi-S: $H(2) = 1.48$, $p = 0.51$; CF-multi-W: $H(2) = 0.371$, $p = 0.831$; Kruskal-Wallis test), the decay time constant (CF-mono: $H(2) = 0.506$, $p = 0.671$; CF-multi-S: $H(2) = 0.17$, $p = 0.931$; CF-multi-W: $H(2) = 2.369$, $p = 0.306$; Kruskal-Wallis test), and the paired-pulse ratio (CF-mono: $H(2) = 0.556$, $p = 0.761$; CF-multi-S: $H(2) = 2.154$, $p = 0.345$; CF-multi-W: $H(2) = 0.523$, $p = 0.769$; Kruskal-Wallis test).

Supplementary Table 4. Electrophysiological Parameters of CF-EPSCs in untransfected PCs, PCs with -NL2 and P/Q-VDCC double KD (NL2 +PQ-DKD), and PCs-with Scrambled NL2 expression and P/Q-VDCC-KD (Scr-NL2 + PQ-KD) (P22-P35)

	CF-group	Number of cells	Amplitude (nA)	10-90% Rise time (ms)	Decay time constant (ms)	CF-PPR (50 ms)
Uninfected	CF-mono	28	2.43 ± 0.95	0.50 ± 0.1	5.98 ± 1.48	0.76 ± 0.04
	CF-multi-S	8	1.96 ± 0.72	0.45 ± 0.12	5.35 ± 0.56	0.79 ± 0.04
	CF-multi-W	8	0.60 ± 0.39	0.48 ± 0.03	3.60 ± 0.58	0.69 ± 0.07
NL2 + PQ - DKD	CF-mono	18	2.23 ± 0.97	0.61 ± 0.18	7.66 ± 2.04*	0.80 ± 0.06
	CF-multi-S	22	1.90 ± 1.07	0.56 ± 0.12	5.86 ± 1.16	0.75 ± 0.17
	CF-multi-W	22	0.45 ± 0.14	0.53 ± 0.15	3.61 ± 2.12	0.64 ± 0.08
Scr-NL2 + PQ-KD	CF-mono	19	2.6 ± 0.96	0.54 ± 0.12	7.44 ± 1.97	0.78 ± 0.08
	CF-multi-S	20	1.98 ± 1.18	0.70 ± 0.12	5.84 ± 1.45	0.79 ± 0.01
	CF-multi-W	20	0.46 ± 0.76	0.60 ± 0.11	4.6 ± 1.61	0.73 ± 0.13

Data were obtained, analyzed, and expressed as shown in Table S3.

There were no significant differences among the three groups of PCs in the amplitude and the paired-pulse ratio for the three types of CF-EPSCs by the Kruskal-Wallis test (Amplitude, CF-mono: H(2) = 2.22, p = 0.33; CF-multi-S: H(2) = 392, p = 0.822; CF-multi-W: H(2) = 1.074, p = 0.585. Paired-pulse ratio, CF-mono: H(2) = 5.316, p = 0.07; CF-multi-S: H(2) = 0.141, p = 0.94; CF-multi-W: H(2) = 3.152, p = 0.207). Significant differences were found among the three groups of PCs by the Kruskal-Wallis test in the 10%-90% rise time for CF-mono (H(2) = 6.12, p = 0.047) and CF-multi-S (H(2) = 6.17, p = 0.0390) but not for CF-multi-W (H(2) = 3.112 p = 0.211). There was also a significant difference in the decay time constant for CF-mono: (H(2) = 8.963, p = 0.011) but not for CF-multi-S (H(2) = 0.861, p = 0.678) or CF-multi-W (H(2) = 1.144, p = 0.564) by the Kruskal-Wallis test. Post hoc analysis using Dunn's test for multiple comparisons revealed a significant difference only in the decay time constant for CF-mono between Uninfested vs NL2+PQ-DKD (p = 0.023).

*$p < 0.05$ by Dunn's test for multiple comparisons.
